# Iridium-Catalysed Transfer Hydrogenation of 1,8-Naphthyridine with Indoline: Access to Functionalized N-Heteroarenes

**DOI:** 10.3390/molecules28237886

**Published:** 2023-12-01

**Authors:** Changjian Zhou, Jiahao Zhang, Yuqing Fu, Chunlian Chen, He Zhao

**Affiliations:** 1 School of Chemistry and Chemical Engineering, Yancheng Institute of Technology, Yancheng 224051, China; zcj@ycit.cn (C.Z.);; 2 Key Lab of Functional Molecular Engineering of Guangdong Province, School of Chemistry & Chemical Engineering, South China University of Technology, Guangzhou 510640, China

**Keywords:** 1,8-naphthyridines, indolines, hydrogen transfer strategy, N-heteroarenes

## Abstract

An iridium-catalysed hydrogen transfer strategy, enabling straightforward access to tetrahydro pyridine derivatives from aryl-1,8-naphthyridines and indolines, was developed. This method proceeds with unprecedented synthetic effectiveness including high step-economic fashion together with the advantages of having no by-product and no need for external high-pressure H_2_ gas, offering an important basis for the transformation of 1,8-naphthyridines and indolines into functionalized products.

## 1. Introduction

Coupling of two components by transfer hydrogenation is an attractive but challenging task in organic chemistry, materials and medicinal science. Its significance lies in the applications in both creation of a wide array of functional products and hydrogen energy storage [1,2,3,4,5,6]. In general, transfer hydrogenation (TH) is a fundamental tool in organic chemistry, to which great efforts have been made because it does not need flammable high-pressure H_2_ gas, and offers more convenient and safe production processes. Pioneered by Benkeser [7,8] and Birch [9,10] reduction, much attention has been focused on the transfer hydrogenation with specific reducing agents [11,12,13,14] and the hydrogenation with high-pressure H_2_ gas [7,8]. Moreover, the Krische group has reported distinguished contributions on the coupling between alcohols and C–C double/triple bonds [15,16,17,18,19,20]. Li and co-workers have demonstrated significant achievements converting phenol derivatives into amines in the presence of NaCO_2_H [21,22] as the hydrogen donor. Despite these valuable contributions, the utilization of such a strategy to construct functionalized N-heterocycles is rarely explored.

**Scheme 1:**
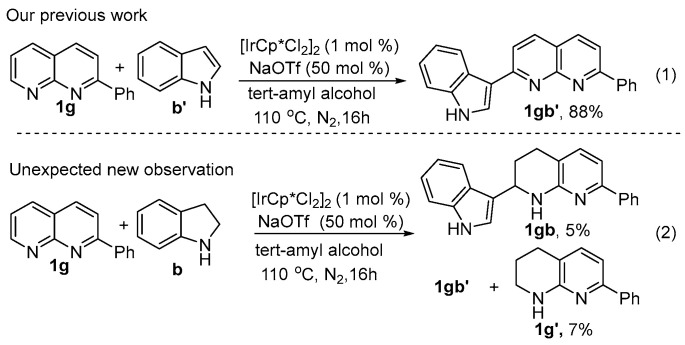
Unexpected New Observation.

1,2,3,4-Tetrahydronaphthyridines (THNADs) constitute the core structure of numerous functionalized molecules, exhibiting diversely interesting biological and therapeutic activities [11,12,13,14,23,24,25]. Traditionally, procedures for these compounds required the use of organometallic hydrides, strong acids or alcohols. However, the preparation of such compounds has to date presented a difficult goal. We have been committed to the ongoing study of N-heterocyclic generation through transfer hydrogenation coupling strategies [26,27,28,29,30,31,32,33,34,35], and we have reported C(sp^3^)-H bond alkylation using tetrahydro-n-heterarene as a coupling partner and hydrogen donor. Initially, our motivation was to test the transfer hydrogenation coupling of indoline with 2-phenyl-1,8-naphthidine. However, after repeated attempts at this reaction, we did not achieve the expected product reported in this paper, and a dehydrogen-coupled compound was detected as the only product at a yield of 12%. Considering that the preparation of indoline feedstock involves the prefabrication step of catalytic hydrogenation of indole derivatives, we then tested the direct coupling of indole with 2-phenyl-1,8-naphthidine under the same conditions. Interestingly, by releasing hydrogen, it produced an even higher yield of 18%. To our knowledge, although a range of approaches have been well explored for functionalization of different sites of the indole and pyrrole skeletons, direct arylation of indole without directing group assistance, prefunctionalization, or consumption of external oxidants remains an unaddressed goal [36,37,38,39,40,41,42,43,44]. After further investigation of these new findings, we reported a new method for directly obtaining nitrogen-containing biological heterocyclic aromatic hydrocarbons by iridium-catalyzed cross-coupling of the β-site of indole/pyrrole with the α-site of N-heterocyclic aromatic hydrocarbons (Scheme 1(1)) [34], we went back and continued to be motivated to test the transfer hydrogenative by using indoline (**b**) as both the coupling partner and the hydrogen donor, coupling with 2-phenyl-1,8-naphthyridine (**1g**) in the presence of iridium NaOTf and tert-amyl alcohol (1.0 mL) at 110 °C for 16 h under N_2_ protection. To our delight, the reaction produced the expected product **1gb** in 5% yield and a tetrahydro-1,8-naphthyridine **1g’** was detected (Scheme 1(2)). Upon a thorough investigation of this observation, we herein report an iridium-catalyzed transfer hydrogenative coupling reaction of indolines and 1,8-naphthyridines, offering a practicable approach for the construction of an α-functionalized tetrahydro-1,8-naphthyridines structurally unique product.

## 2. Results and Discussion

Our initial studies focused on developing a more efficient catalytic system for the coupling of 2-(4-(trifluoromethyl)phenyl)-1,8-naphthyridine **1a** with 2-methylindoline **2a** as a model system. First, the reaction was performed at 130 °C for 16 h by using [Cp*IrCl_2_]_2_ (1 mol %) as a catalyst and NaOTf (50 mol %) as an additive, which afforded the product 3aa in 35% yield (entry 1). Then, a series of acids and bases were evaluated (50 mol %, entries 2–7); unfortunately, they were totally ineffective or less effective. Gratifyingly, no use of additive led to an improved yield (entry 9, 68%), and the absence of catalyst failed to yield any product (entry 10), indicating that the iridium catalyst plays a crucial role in affording the product. Further, serval ligands (entries 12–13) did not show any activity under the studied reaction conditions. Moreover, other palladium and iridium catalysts employed for the reaction showed less reactivity for the transformation. Finally, change of reaction temperatures (entry 10) or solvents (entries 14–15) were not fruitful since no increase of product yield was obtained. Thus, the optimal conditions are as indicated in entry 9 of Table 1.

**Scheme 2:**
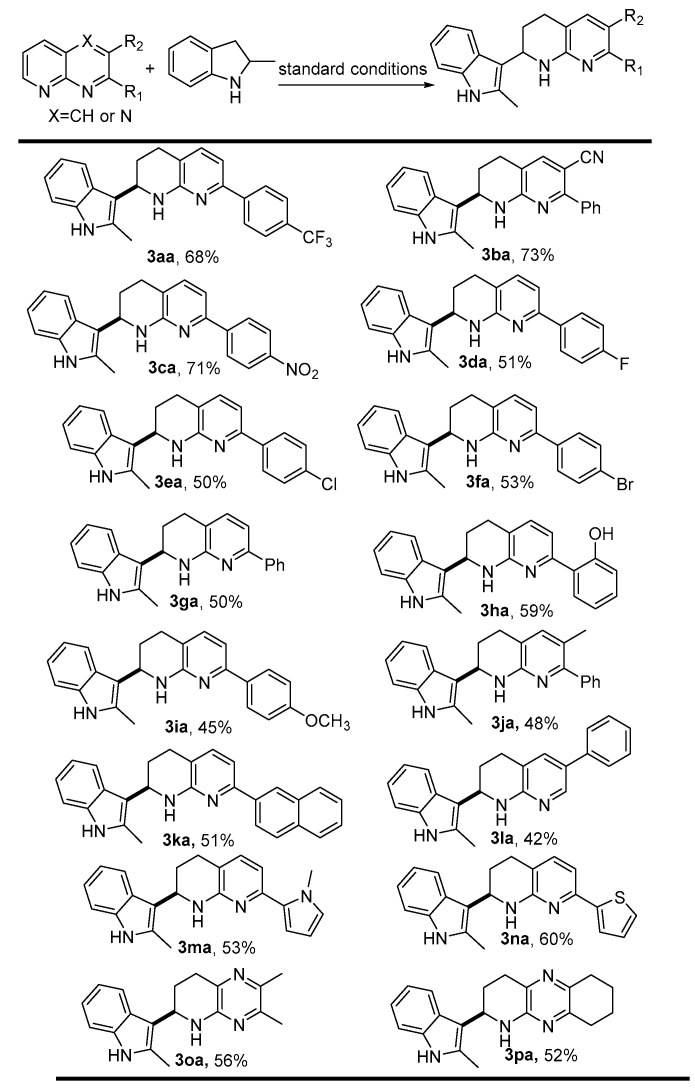
Variations of 1,8-nphthyridines. Standard conditions: all the reaction in *t*-amyl alcohol (1.0 mL) was performed with **1** (0.2 mmol), **2** (0.3 mmol), [Cp*IrCl_2_]_2_ (1 mol %), at 110 °C for 16 h under N_2_ protection.

With the optimal conditions in hand, we next examined the generality and the limitation of the synthetic protocol. First, a series of 1,8-nphthyridines [**1a**-**1l**] and N-heteroaromatics (**1m**-**1p**) with 2-methylindoline **2a**, for their structures (see Supporting Information (SI) Table S1) were tested. As shown in Scheme 2, all the reactions proceeded smoothly and furnished the desired products in moderate to good isolated yields. The results indicated that the substituents on the aryl ring of reactant 1 significantly affected the reactions. Specially, electron-withdrawing substitutents afforded the products (**3aa**-**ca**) in much higher yields than those of electro-rich substitutents. This observation might be attributed to the electron-withdrawing groups that could enhance the electrophilicity of the in situ formed imine intermediate, thus favoring the coupling process. Gratifyingly, 2-(1-methyl-1*H*-pyrrol-2-yl)-1,8-naphthyridine (**1m**) proved to be an effective coupling partner, yielding the products in reasonable yields (see **3ma**). Moreover, Substrate **1o** and **2p**, nitrogen-modified 1,8-naphthyridines, effectively coupled with **1a** to give compound **3oa** and **3pa** in 56% and 52% yield, respectively; this example demonstrates the potential of the methodology to be applied to other heterocyclic scaffolds. It is worth mentioning that a series of functional groups such as -CF_3_, OH, -Cl, -Br, -NO_2_, and -CN are well tolerated in the synthetic protocol which would offer the potential for molecular complexity via further chemical transformation.

**Scheme 3:**
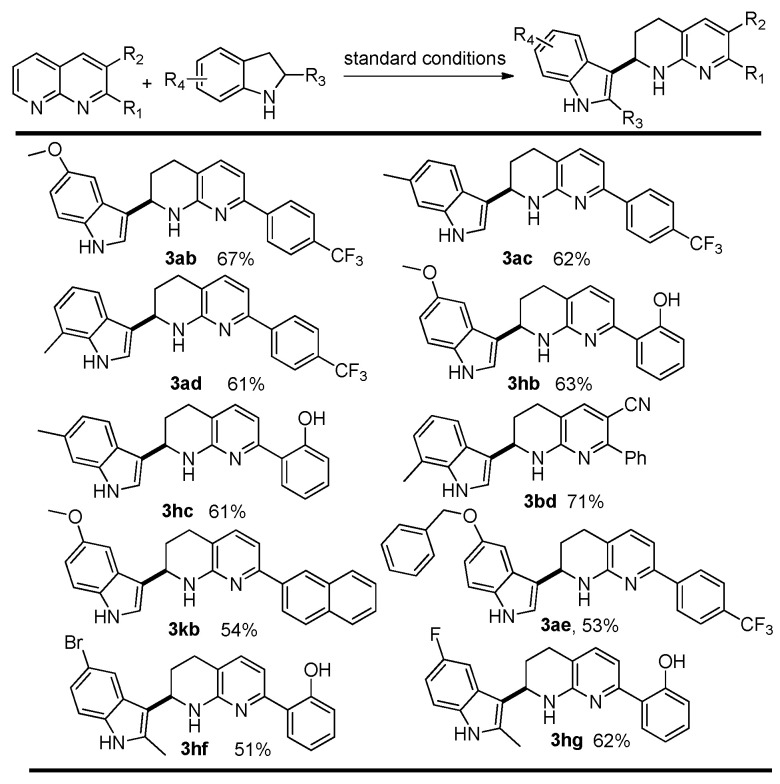
Variation of indolines.

Subsequently, we focused on the variation of both coupling partners. Thus, various combinations of **1** with indolines **2** were tested. Similar to the results described in Scheme 2, all the reactions afforded the desired products in moderate-to-excellent isolated yields (Scheme 3). Gratifyingly, a series of indolines underwent efficient transfer hydrogen evolution cross-coupling reactions, and the reactions of electron-rich indolines (**2b**-**c**) with electron-poor 1,8-nphthyridines (**1a**,**1h**) could give satisfactory yields, presumably because the electron-donating group could enhance the nucleophilicity of the indole skeleton, which is also beneficial for the coupling step.

**Scheme 4:**
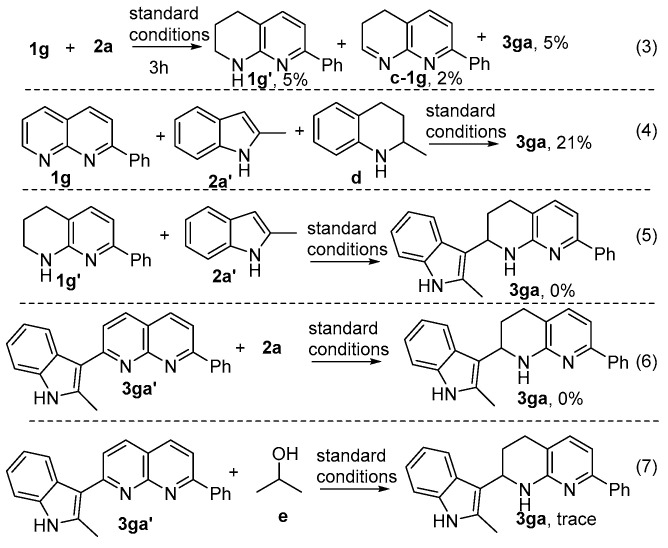
Control Experiments.

To gain insights into the possible mechanism, several verification experiments were performed. The model reaction was subsequently interrupted after 3 h to analyse the intermediates. By means of GC analyses, product **3ga**, tetra- and di-hydronaphthyridine were detected in 5%, 5% and 2% yields, respectively (Scheme 4). Then, the reaction **1g’**, **2a’** and 2-methyl-1,2,3,4-tetrahydroquinoline as the hydrogen donor under standard conditions produced product 3aa in 21% yield. Further it was found that **2a’** failed to directly couple with the di-hydronaphthyridine **1g’** to give product **3ga**, indicating that tetrahydronaphthyridine **1g’** is not the reaction intermediate; finally, treating **3ga’** with equimolar amount of **2a** or e was not able to afford **3ga** (Scheme 4), showing that **c-1g** may be the key intermediate and **3ga’** as the intermediate of the reaction is less likely.

**Scheme 5:**
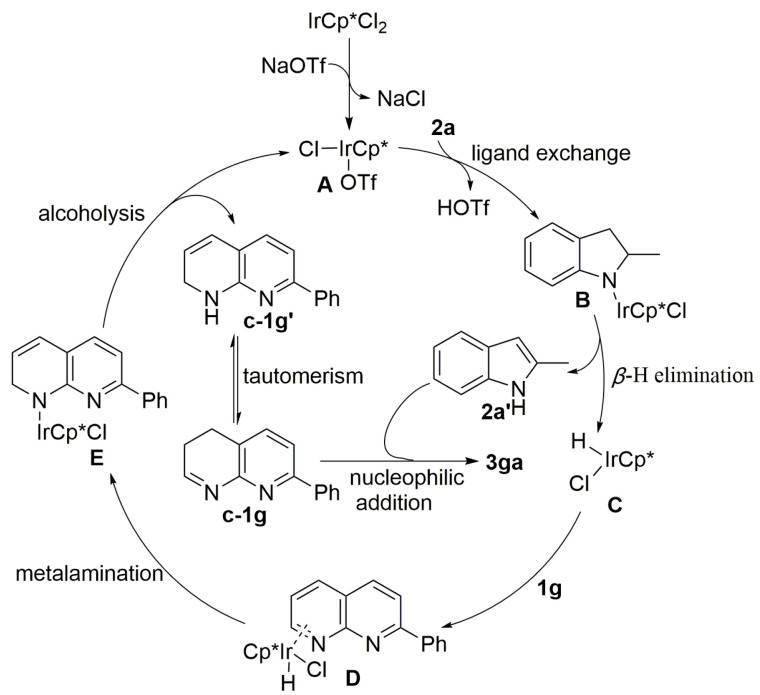
Plausible Catalytic Cycle.

Although the mechanism of this reaction has not been fully elucidated, on the basis of the above-observed findings, a hydrogen transfer is proposed in Scheme 5. Based on metal-catalyzed transfer hydrogen mechanism reported in the literature [45,46] and the above control experiments, a plausible reaction pathway is proposed in Scheme 5. First, IrCp*Cl_2_ and NaOTf proceed in a ligand exchange process to generate complex **A** which thereupon reacts with 2-methylindoline **2a** to give **B** followed by β-H elimination to form 2-methyl-1*H*-indole **2a’** and metal hydride **C**. Next, 2-phenyl-1,8-naphthyridine **1g** undergoes coordination with **C** and then experiences hydrometallation to afford intermediate **E**. Subsequently, with the alcoholysis of E, transfer hydrogenation intermediate **c-1g** or its tautomerism **c-1g’** is produced, and **A** is regenerated to accomplish the catalytic cycle. Finally, the formed **c-1g** and 2-methyl-1*H*-indole **2a’** undergoes the classic nucleophilic addition to provide the desired tetrahydro α-functionalized product **3ga**.

## 3. Experimental

### 3.1. General Information

All the obtained products were characterized by melting points (m.p.), ^1^H-NMR, ^13^C-NMR and infrared spectra (IR). Melting points were measured on an Electrothemal W-X4 microscopy digital melting point apparatus and are uncorrected; IR spectra were recorded on a FTLA2000 spectrometer; 1H-NMR and ^13^C-NMR spectra were obtained on Bruker-400 and referenced to CHCl_3_ (7.26 ppm for ^1^H, and 77.2 ppm for ^13^C) or DMSO-*d_6_* (2.50 ppm for ^1^H, and 39.5 ppm for ^13^C). Chemical shifts were reported in parts per million (ppm, δ) downfield from tetramethylsilane. Proton coupling patterns are described as singlet (s), doublet (d), triplet (t), multiplet (m); TLC was performed using commercially prepared 100–400 mesh silica gel plates (GF254), and visualization was effected at 254 nm; Unless otherwise stated, all the reagents were purchased from commercial sources (J&KChemic, TCI, Fluka, Acros, SCRC, Shanghai, China), used without further purification.

### 3.2. Substrates Preparation

The preparation of 1,8-naphthyridines **2**. 2-aminonicotinaldehyde **4** (5 mmol), ketones **5** (5 mmol), *t*-BuOK (20 mol %) and ethanol (10 mL) were introduced in a flask (50 mL). Then, it was stirred at 50 °C under atmosphere for 2 h. After cooling down to room temperature, the resulting mixture was filtered and washed with ethyl acetate, and then concentrated by removing the solvent under vacuum. Finally the residue was purified by preparative TLC on silica, eluting with petroleum ether (60–90 °C): ethyl acetate (10:1, *v*/*v*) to give 1,8-naphthyridines, all the substrates used in our reaction are listed in Table S1. All the reagents were purchased from Bide Pharmatech Ltd. and Energy Chemical, all the solvents were purchased from Greagent (Shanghai Titansci incorporated company, Shanghai, China) and used without further purification. All the reactions were heated by metal sand bath (WATTCAS, LAB-500, https://www.wattcas.com (accessed on 17 May 2017)).

### 3.3. Typical Procedure for the Synthesis of Ester ***3aa***

The mixture of 2-phenyl-1,8-naphthyridine **1a** (0.2 mmol), 2-methylindoline **2a** (0.3 mmol), [Cp*IrCl_2_]_2_ and *t*-amyl alcohol (1.0 mL) were added to the Schlenk tube (50 mL) successively under nitrogen protection; then, the reaction tube was closed and placed in an oil bath at a temperature of 110 °C, where the reaction took place for 16 h. After that, the Schlenk tube was then removed from the oil bath and placed in the air to cool. After cooling down to room temperature, the reaction mixture was concentrated by removing the solvent under vacuum. Finally, the residue was purified by preparative TLC on silica, eluting with ethyl acetate: petroleum ether (60–90 °C) = 1:5, to give the desired product **3aa**.

## 4. Conclusions

In summary, by employing hydrogen transfer strategy and indoline as both hydrogen donor and the reactant, we have developed a novel straightforward synthesis of functionalized N-heterocycles. This method proceeds with unprecedented synthetic effects including high step-economic fashion together with the advantages of being without any by-product and having no need for external high pressure H_2_, offering a practicable approach for the construction of an α-functionalized tetrahydro-1,8-naphthyridines structurally unique product. Further investigation applying the hydrogen transfer coupling strategy in other hetero cyclic systems as well as the asymmetric synthesis is ongoing in our laboratory and will be reported in due course.

## Figures and Tables

**Table 1:**
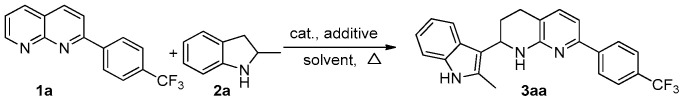
Screening of the Optimal Conditions ^a^.

**Entry**	**Catalyst (mol %)**	**Additive (mol %)**	**3aa yield%** **^b^**
1	[Cp*IrCl_2_]_2_ (1)	NaOTf (10)	35
2	[Cp*IrCl_2_]_2_ (1)	benzoic acid (50)	27
3	[Cp*IrCl_2_]_2_ (1)	CF_3_COOH (50)	29
4	[Cp*IrCl_2_]_2_ (1)	HOTf (50)	25
5	[Cp*IrCl_2_]_2_ (1)	Et_3_N (50)	23
6	[Cp*IrCl_2_]_2_ (1)	DBU (50)	-
7	[Cp*IrCl_2_]_2_ (1)	NaOH (50)	-
8	[Cp*IrCl_2_]_2_ (1)	-	59
9	[Cp*IrCl_2_]_2_ (1)		68 ^c^
10	[Cp*IrCl_2_]_2_ (1)	-	(39, 47) ^d^
11	-	-	-
12	[Cp*IrCl_2_]_2_ (1)	DPPB	-
13	[Cp*IrCl_2_]_2_ (1)	1,10-Phenanthroline	-
14	[Cp*IrCl_2_]_2_ (1)	-	(51, 55) ^e^
15	[Cp*IrCl_2_]_2_ (1)	-	(47, trace) ^f^
16	[IrClCOD]_2_ (1)	-	trace
17	Pd(OAc)_2_ (5)	-	17%

^a^ Reaction conditions: unless otherwise stated, the reaction in *t*-amyl alcohol (1.0 mL) was performed with **1a** (0.2 mmol), **2a** (0.2 mmol), catalyst (1 mol %), additive (50 mol %) at 130 °C for 16 h under N_2_ protection. ^b^ Isolated yield. ^c^ 2-methylindoline **2a** (0.3 mmol). ^d^ Temperature at 100 °C and 150 °C. ^e^ Yields are with respect to p-xylene and 4-chlorobenzene as the solvents, respectively. ^f^ Yields are with respect to 1,4-dioxane and DMF as the solvents, respectively. Cp* = C_5_Me_5_. DPPB = butane-1,4-diylbis[diphenylphosphine].

## Data Availability

Data are contained within the article and Supplementary Materials.

## Supplementary Materials

The following supporting information can be downloaded at: https://www.mdpi.com/article/10.3390/molecules28237886/s1, Table S1: Synthesis of substrates 1,8-naphthyridines; Scheme S1: Substrates employed. Refs. [47,48,49,50,51,52,53] are cited in Supplementary Materials.
