# Supplementary material for: Iridium-Catalysed Transfer Hydrogenation of 1,8-Naphthyridine with Indoline: Access to Functionalized N-Heteroarenes
Source: Molecules. 2023 Dec 1;28(23):7886. doi: 10.3390/molecules28237886 (PMC10707882; doi:10.3390/molecules28237886)

Supporting Information

Iridium-Catalysed Transfer Hydrogenation of 1,8-Naphthyridine with Indoline: Access to Functionalized N-Heteroarenes

Changjian Zhou [1,†], Jiahao Zhang [1,†], Yuqing Fu [1], Chunlian Chen [2] and He Zhao [1,*]

[1] School of Chemistry and Chemical Engineering, Yancheng Institute of Technology, Yancheng 224051, China; zcj@ycit.cn (C.Z.)
[2] Key Lab of Functional Molecular Engineering of Guangdong Province, School of Chemistry & Chemical Engineering, South China University of Technology, Guangzhou 510640, China
* Correspondence: zhaohe@ycit.edu.cn
† These authors contributed equally to this work.

Table of contents

General information	S1
Substrates preparation	S1
Typical procedure for the synthesis of product **3aa**	S2
Substrates employed	S3
Analytic data of the obtained compounds	S3–S16
NMR spectra of obtained compounds	S17–S42

General information

All the obtained products were characterized by melting points (m.p.), ^{1}H-NMR, ^{13}C-NMR and infrared spectra (IR). Melting points were measured on an Electrothemal W-X4 microscopy digital melting point apparatus and are uncorrected; IR spectra were recorded on a FTLA2000 spectrometer; ^{1}H-NMR and ^{13}C-NMR spectra were obtained on Bruker-400 and referenced to $CHCl_3$ (7.26 ppm for ^{1}H, and 77.2 ppm for ^{13}C) or DMSO-d_6 (2.50 ppm for ^{1}H, and 39.5 ppm for ^{13}C). Chemical shifts were reported in parts per million (ppm, δ) downfield from tetramethylsilane. Proton coupling patterns are described as singlet (s), doublet (d), triplet (t), multiplet (m); TLC was performed using commercially prepared 100-400 mesh silica gel plates (GF254), and visualization was effected at 254 nm; Unless otherwise stated, all the reagents were purchased from commercial sources (J&KChemic, TCI, Fluka, Acros, SCRC), used without further purification.

Substrates preparation

The preparetion of substrates was similar to the literature reports [].The preparation of 1,8-naphthyridines **2**. 2-aminonicotinaldehyde **4** (5 mmol), ketones **5** (5 mmol), *t*-BuOK (20 mol %) and ethanol (10 mL) were introduced in a flask (50 mL). Then, it was stirred at 50 °C under atmosphere for 2 hours. After cooling down to room temperature, the reaction mixture was concentrated by removing the solvent under vacuum, and the residue was purified by column chromatography.

Table S1. Synthesis of substrates 1,8-naphthyridines

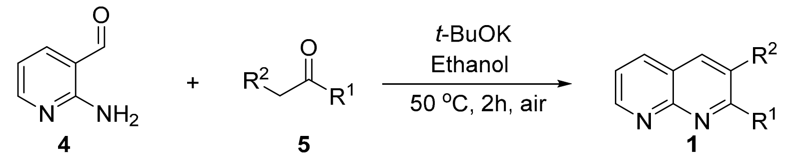

Entry	5		1
1	R^1=4-CF_3Ph	R^2=H	**1a**
2	R^1=Ph	R^2=CN	**1b**
3	R^1=4-NO_2Ph	R^2=H	**1c**

4	R^1=4-FPh	R^2=H	**1d**
5	R^1=4-ClPh	R^2=H	**1e**
6	R^1=4-BrPh	R^2=H	**1f**
7	R^1=Ph	R^2=H	**1g**
8	R^1=2-HOPh	R^2=H	**1h**
9	R^1=4-OMePh	R^2=H	**1i**
10	R^1=Ph	R^2=Me	**1j**
11	R^1=Naphth	R^2=H	**1k**
12	R^1= H	R^2=Ph	**1l**
13	R^1= 1-methyl-1H-pyrrole	R^2=H	**1m**
14	R^1=thiophen-2-yl	R^2=H	**1n**

Reference

[1] S. A. Moya. Appl. Organomet. Chem., 2008, 22, 471-478.

[2] B. Xiong, S. d. Zhang, F. H. Jiang, M. Zhang. Org. Lett., 2016, 18 (4), 724 − 727.

[3] E. M. Hawes, D. K. J. Gorecki, R. G. Gedir. J. Med. Chem., 1977, 20(6): 838-841.

[4] B. Sreenivasulu, K. V. Reddy. Current Science, 1977, 46(17): 597-598.

[5] K. Mogilaiah, Indian J. Chem., 2010, 49B(2), 253-255.

[6] P. Galatsis, K. Yamagata, J. A. Wendt. Bioorg. Med. Chem. Lett., 2007, 17(23): 6525-6528.

[7] X. W. Chen, H. Zhao, B. Xiong, H. F. Jiang, P. H. Dixneuf, M. Zhang. Org. Biomol. Chem., 2017, 15, 6093-6097.

[8] X. W. Chen, H. Zhao, C. L. Chen, H. F. Jiang, M. Zhang. Angew. Chem. Int. Ed. 2017, 56, 14232-14236.

Typical procedure for the synthesis of product 3aa

Under N_2 atmosphere, 2-phenyl-1,8-naphthyridine **1a** (0.2 mmol), 2-methylindoline **2a** (0.3 mmol), $[Cp^*IrCl_2]_2$ and *t*-amyl alcohol (1.0 mL) were introduced in a Schlenk tube (50 mL), successively. Then, the Schlenk tube was closed and the resulting

mixture was stirred at 110 °C for 16 h. After cooling down to room temperature, the reaction mixture was concentrated by removing the solvent under vacuum. Finally, the residue was purified by preparative TLC on silica, eluting with ethyl acetate : petroleum ether = 1 : 5, to give the desired product **3aa**.

Scheme S1. Substrates employed

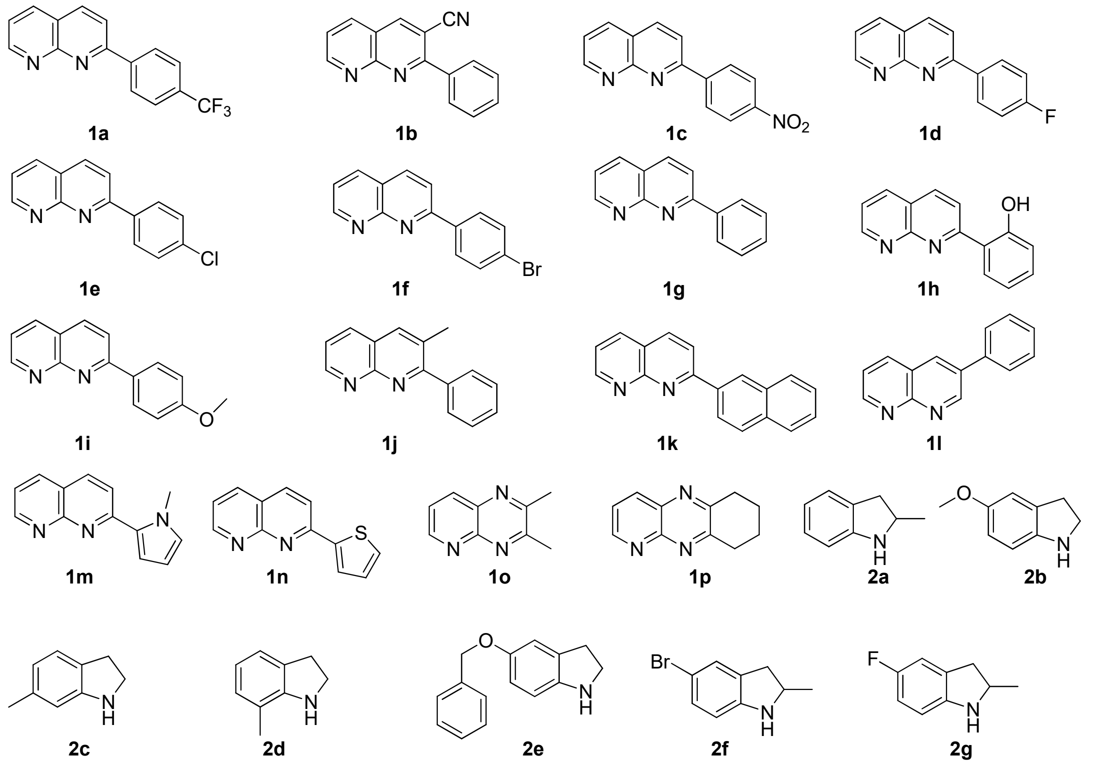

Analytic data of the obtained compounds

(1) 2-(2-methyl-1H-indol-3-yl)-7-(4-(trifluoromethyl)phenyl)-1,2,3,4-tetrahydro-1,8-naphthyridine (**3aa**)

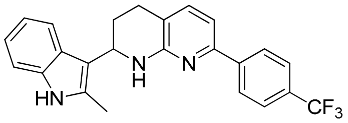

Yellow solid, (48.03 mg, 59% yield), m.p.: 172 - 173 °C; ^{1}H NMR (400 MHz, $CDCl_3$): δ 7.88 (d, J = 8.1 Hz, 2H), 7.83 (s, 1H), 7.53 (d, J = 7.9 Hz, 1H), 7.49 (d, J = 8.1 Hz, 2H), 7.17 (d, J = 7.5 Hz, 1H), 7.07 (d, J = 6.8 Hz, 1H), 6.97 (t, J = 7.5 Hz, 1H), 6.91 - 6.85 (m, 2H), 5.15 (s, 1H), 4.70 (dd, J = 10.6, 2.6 Hz, 1H), 2.88 - 2.76 (m, 1H), 2.74 -

2. 66 (m, 1H), 2.34 - 2.20 (m, 1H), 2.18 (d, J = 9.5 Hz, 3H), 1.94 - 1.85 (m, 1H). ^{13}C NMR (101 MHz, $CDCl_3$) δ 156.65 (s), 152.26 (s), 143.30 (s), 136.97 (s), 135.40 (s), 131.85 (s), 129.91 (q, J = 32.2 Hz), 126.88 (s), 125.85 (s), 125.42 (q, J = 3.6 Hz), 123.14 (s), 121.24 (s), 119.44 (s), 119.25 (s), 115.99 (s), 112.47 (s), 110.54 (s), 110.08 (s).IR (KBr): 3428, 2358, 1609, 1460, 1166, 1120, 1069, 1011, 811, 744 cm^{-1}. HRMS (ESI): Calcd. for $C_{24}H_{21}F_3N_3$ $[M+H]^+$: 408.1682; found: 408.1685.

(2) 7-(2-methyl-1H-indol-3-yl)-2-phenyl-5,6,7,8-tetrahydro-1,8-naphthyridine-3-carbonitrile (**3ba**)

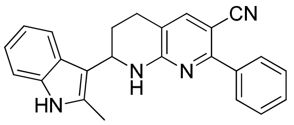

Yellow solid, (51.69 mg, 71% yield), m.p.: 204 - 205 °C; ^{1}H NMR (400 MHz, $CDCl_3$): δ 7.99 (s, 1H), 7.90 - 7.80 (m, 2H), 7.57 (d, J = 7.7 Hz, 1H), 7.50 (s, 1H), 7.48 - 7.40 (m, 3H), 7.28 (d, J = 8.0 Hz, 1H), 7.12 (t, J = 7.5 Hz, 1H), 7.07 - 6.09 (m, 1H), 5.77 (s, 1H), 4.92 (dd, J = 10.5, 3.3 Hz, 1H), 3.00 - 2.81 (m, 2H), 2.39 (s, 3H), 2.38 - 2.28 (m, 1H), 2.15 - 2.08 (m, 1H). ^{13}C NMR (101 MHz, $CDCl_3$): δ 139.89, 135.32, 131.95, 129.61, 128.58, 128.44, 126.45, 121.51, 119.70, 118.81, 111.26, 110.58, 94.17, 49.72, 27.70, 26.29, 12.06. IR (KBr): 3056, 2924, 2356, 2212, 1702, 1602, 1508, 1434, 922, 742, 698 cm^{-1}. HRMS (ESI): Calcd. for $C_{24}H_{21}N_4$ $[M+H]^+$: 365.1761; found: 365.1759.

(3) 2-(2-methyl-1H-indol-3-yl)-7-(4-nitrophenyl)-1,2,3,4-tetrahydro-1,8-naphthyridine (**3ca**)

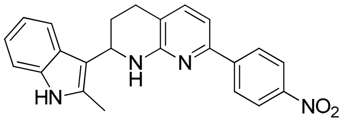

Yellow solid, (44.54 mg, 58% yield), m.p.: 180 - 181 °C; ^{1}H NMR (400 MHz, $CDCl_3$): δ 8.19 (d, J = 9.6 Hz, 2H), 8.07 (d, J = 8.8 Hz, 2H), 7.96 (s, 1H), 7.67 (d, J = 8.0 Hz, 1H), 7.35 (d, J = 7.6 Hz, 1H), 7.27 (d, J = 8.0 Hz, 1H), 7.14 (t, J = 7.6 Hz, 1H), 7.07 – 7.03 (m, 2H), 5.45 (s, 1H), 4.80 (dd, J = 10.8, 3.2 Hz, 1H), 3.02 – 2.86 (m, 2H), 2.46 – 2.41 (m, 1H), 2.38 (s, 3H), 2.10 - 2.07 (m, 1H). ^{13}C NMR (101 MHz,

CDCl3) δ: 156.72, 150.95, 147.39, 145.91, 136.81, 135.34, 131.77, 127.04, 126.82, 123.69, 121.25, 119.41, 119.15, 116.80, 112.37, 110.52, 110.37, 49.44, 28.37, 26.91, 11.97. IR (KBr): 3120, 2358, 1667, 1653, 1531, 1371, 1266, 825, 764cm^{-1}. HRMS (ESI): Calcd. for $C_{23}H_{21}N_4O_2$ $[M+H]^+$: 385.1659; found: 385.1622.

(4) 7-(4-fluorophenyl)-2-(2-methyl-1H-indol-3-yl)-1,2,3,4-tetrahydro-1,8-naphthyrid-ne (**3da**)

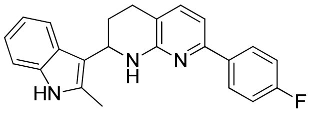

Yellow solid, (31.42 mg, 44% yield), m.p.: 123 - 124 °C; ^{1}H NMR (400 MHz, $CDCl_3$): δ 7.88 - 7.74 (m, 3H), 7.56 (d, *J* = 7.8 Hz, 1H), 7.18 (d, *J* = 7.4 Hz, 1H), 7.10 (s, 1H), 7.01 - 6.88 (m, 4H), 6.83 (d, *J* = 7.4 Hz, 1H), 5.03 (s, 1H), 4.74 (dd, *J* = 10.6, 2.8 Hz, 1H), 2.89 - 2.79 (m, 1H), 2.72 (d, *J* = 16.2 Hz, 1H), 2.32 - 2.27 (m, 1H), 2.23 (s, 3H), 1.93 (d, *J* = 9.9 Hz, 1H). ^{13}C NMR (101 MHz, $CDCl_3$) δ 163.09 (d, J = 247.0 Hz), 156.43 (s), 152.92 (s), 136.97 (s), 136.06 (s), 135.35 (s), 131.76 (s), 128.37 (d, J = 8.1 Hz), 126.92 (s), 121.22 (s), 119.42 (s), 119.29 (s), 115.32 (d, J = 21.4 Hz), 114.73 (s), 112.60 (s), 110.46 (s), 109.39 (s), 49.52 (s), 28.62 (s), 26.79 (s), 12.03 (s). IR (KBr): 3060, 1698, 1591, 1520, 1459, 1230, 807, 742, 674 cm^{-1}. HRMS (ESI): Calcd. for $C_{23}H_{21}FN_3$ $[M+H]^+$: 358.1714; found: 358.1719.

(5) 7-(4-chlorophenyl)-2-(2-methyl-1H-indol-3-yl)-1,2,3,4-tetrahydro-1,8-naphthyrid-ine (**3ea**)

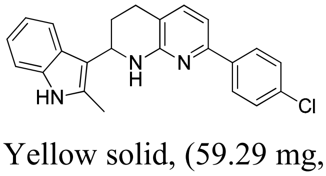

Yellow solid, (59.29 mg, 81% yield), m.p: 163 - 164 °C; ^{1}H NMR (400 MHz, $CDCl_3$) δ 7.88 – 7.74 (m, 3H), 7.56 (d, *J* = 7.8 Hz, 1H), 7.18 (d, *J* = 7.4 Hz, 1H), 7.10 (s, 1H), 7.01 – 6.88 (m, 4H), 6.83 (d, *J* = 7.4 Hz, 1H), 5.03 (s, 1H), 4.74 (dd, *J* = 10.6, 2.8 Hz, 1H), 2.89 – 2.79 (m, 1H), 2.72 (d, *J* = 16.2 Hz, 1H), 2.30 (dd, *J* = 12.2, 4.5 Hz, 1H), 2.23 (s, 3H), 1.93 (d, *J* = 9.9 Hz, 1H). ^{13}C NMR (101 MHz, $CDCl_3$) δ 164.32, 161.86, 156.43, 152.92, 136.97, 136.06, 135.35, 131.76, 128.41, 128.33, 126.91, 121.22,

119.41, 119.29, 115.42, 115.21, 114.73, 112.60, 110.46, 109.38, 49.52, 28.61, 26.79, 12.03. IR (KBr): 3060, 1653, 1459, 1230, 1145, 825, 764 cm^{-1}. HRMS (ESI): Calcd. for $C_{23}H_{21}ClN_3$ [M+H]$^+$: 367.1194 ; found: 367.1190.

(6) 7-(4-bromophenyl)-2-(2-methyl-1H-indol-3-yl)-1,2,3,4-tetrahydro-1,8-naphthyridine (**3fa**)

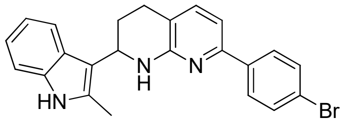

Yellow solid, (39.20 mg, 47% yield), m.p.: 164 - 165 °C; 1H NMR (400 MHz, $CDCl_3$): δ 7.78 (d, J = 8.1 Hz, 3H), 7.59 (d, J = 7.6 Hz, 1H), 7.29 – 7.14 (m, 4H), 7.02 (t, *J* = 7.2 Hz, 1H), 6.98 – 6.91 (m, 1H), 6.88 (d, *J* = 7.3 Hz, 1H), 5.05 (s, 1H), 4.78 (d, *J* = 9.0 Hz, 1H), 2.95 – 2.82 (m, 1H), 2.76 (d, *J* = 15.8 Hz, 1H), 2.30 (s, 4H), 1.97 (d, *J* = 10.6 Hz, 1H). ^{13}C NMR (101 MHz, $CDCl_3$): δ 156.43, 152.65, 138.38, 136.87, 135.32, 134.09, 131.66, 128.59, 127.88, 126.90, 121.27, 119.45, 119.30, 115.07, 112.67, 110.39, 109.44, 49.50, 28.58, 26.81, 12.09. IR (KBr): 2926, 2358, 1667, 1602, 1371, 1266, 809, 742 cm^{-1}. HRMS (ESI): Calcd. for $C_{23}H_{21}BrN_3$ [M+H]+: 418.913; found：418.0911.

(7) 2-(2-methyl-1H-indol-3-yl)-7-phenyl-1,2,3,4-tetrahydro-1,8-naphthyridine (**3ga**)

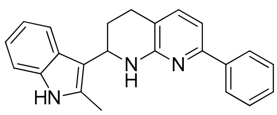

Brown solid, (23.73 mg, 35% yield), m.p.: 138 - 139 °C; H NMR (400 MHz, $CDCl_3$): δ 7.83 (d, *J* = 7.4 Hz, 3H), 7.57 (d, *J* = 7.9 Hz, 1H), 7.32 - 7.27 (m, 2H), 7.22 (dd, *J* = 10.0, 7.4 Hz, 2H), 7.13 - 7.07 (m, 1H), 6.99 (t, *J* = 7.1 Hz, 1H), 6.92 (m, *J* = Hz, 2H), 5.00 (s, 1H), 4.75 (dd, *J* = 10.7, 3.0 Hz, 1H), 2.95 - 2.79 (m, 1H), 2.78 - 2.66 (m, 1H), 2.38 - 2.25 (m, 1H), 2.23 (s, 3H), 2.04 - 1.75 (m, 1H). ^{13}C NMR (101 MHz, $CDCl_3$): δ 156.48, 154.06, 140.04, 136.91, 135.35, 131.75, 128.54, 128.23, 126.95, 126.70, 121.20, 119.40, 119.32, 114.75, 112.68, 110.44, 109.81, 49.52, 28.66, 26.84, 12.05. IR (KBr): 3057, 2924, 1665, 1598, 1455, 1372, 1302, 1117, 750, 695 cm^{-1}. HRMS (ESI) : Calcd. for $C_{23}H_{22}N_3$ [M+H]$^+$: 340.1808; found: 340.1813.

(8) 2-(7-(2-methyl-1H-indol-3-yl)-5,6,7,8-tetrahydro-1,8-naphthyridin-2-yl)phenol (**3ha**)

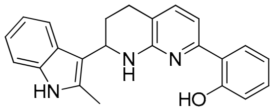

Yellow solid, (37.63 mg, 53% yield), m.p.: 120 - 121 °C; ^{1}H NMR (400 MHz, DMSO) δ 14.25 (s, 1H), 10.87 (s, 1H), 7.84 (d, *J* = 7.8 Hz, 1H), 7.47 (d, *J* = 7.8 Hz, 2H), 7.42 (d, *J* = 7.7 Hz, 1H), 7.29 (d, m, 1H), 7.23 - 7.16 (m, 2H), 7.03 - 6.96 (m, 1H), 6.92 - 6.86 (m, 1H), 6.85 - 6.79 (m, 2H), 4.89 (dd, *J* = 9.5, 2.9 Hz, 1H), 2.97 – 2.85 (m, 1H), 2.76 (d, *J* = 16.2 Hz, 1H), 2.39 (s, 3H), 2.25 - 2.14(m, 1H), 2.04 - 1.93 (m, 1H). ^{13}C NMR (101 MHz, DMSO): δ 159.36, 154.47, 153.33, 137.71, 135.74, 132.58, 130.54, 127.27, 126.73, 120.44, 119.90, 118.97, 118.72, 118.18, 114.70, 112.14, 111.05, 106.78, 48.73, 28.50, 26.32, 12.13. IR (KBr): 3419, 2926, 1514, 1469, 1354, 1279, 1118, 747 cm^{-1}; HRMS (ESI): Calcd. for $C_{23}H_{22}N_3O$ [M+H]$^+$: 356.1757; found: 356.1755.

(9) 2-(1H-indol-3-yl)-7-(4-methoxyphenyl)-1,8-naphthyridine (**3ia**)

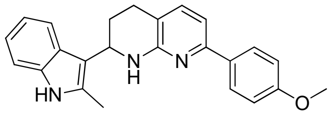

Yellow solid, (29.52 mg, 40% yield), m.p.: 160 - 161 °C; ^{1}H NMR (400 MHz, $CDCl_3$): δ 7.90 (s, 1H), 7.78 (d, *J* = 8.6 Hz, 2H), 7.57 (d, *J* = 7.9 Hz, 1H), 7.17 (d, *J* = 7.5 Hz, 1H), 7.10 (d, *J* = 7.0 Hz, 1H), 6.98 (t, *J* = 7.4 Hz, 1H), 6.91 (t, *J* = 7.5 Hz, 1H), 6.87 – 6.79 (m, 3H), 4.98 (s, 1H), 4.73 (dd, *J* = 10.7, 3.0 Hz, 1H), 3.70 (s, 3H), 2.88 – 2.78 (m, 1H), 2.75 – 2.66 (m, 1H), 2.35 – 2.24 (m, 1H), 2.22 (s, 3H), 1.97 – 1.87 (m, 1H). ^{13}C NMR (101 MHz, $CDCl_3$): δ 159.89, 156.39, 153.72, 136.91, 135.36, 132.73, 131.76, 127.88, 126.96, 121.16, 119.36, 119.32, 113.95, 113.91, 112.72, 110.43, 109.06, 77.45, 77.14, 76.82, 55.36, 49.52, 28.73, 26.79, 12.03. IR (KBr):2924, 2750, 1597, 1502, 1456, 1249, 1026, 806, 743, 676 cm^{-1}. HRMS (ESI): Calcd. for $C_{24}H_{24}N_3O$ [M+H]$^+$: 370.1914; found: 370.1919.

(10)6-methyl-2-(2-methyl-1H-indol-3-yl)-7-phenyl-1,2,3,4-tetrahydro-1,8-naphthyridin-e (**3ja**)

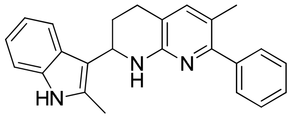

Yellow solid, (31.77 mg, 45% yield), m.p.: 144 - 145 °C ^{1}H NMR (400 MHz, $CDCl_3$): δ 8.42 (s, 1H), 7.76 (d, J = 7.4 Hz, 1H), 7.67 – 7.59 (m, 2H), 7.48 (t, J = 7.2 Hz, 2H), 7.41 (t, J = 6.9 Hz, 1H), 7.28 (s, 1H), 7.23 (d, J = 7.8 Hz, 1H), 5.01 (s, 1H), 4.89 (d, J = 10.2 Hz, 1H), 3.12 – 2.99 (m, 1H), 2.92 (d, J = 15.9 Hz, 1H), 2.45 (dd, J = 20.7, 10.2 Hz, 1H), 2.32 (s, 3H), 2.30 (s, 3H), 2.12 m, 1H). ^{13}C NMR (101 MHz, $CDCl_3$): δ 154.71, 154.34, 141.15, 139.40, 135.37, 131.91, 129.10, 128.11, 127.50, 126.97, 120.98, 119.20, 119.00, 115.01, 112.50, 110.53, 49.63, 28.86, 26.75, 18.93, 11.94. IR (KBr): 3060, 2750, 1698, 1591, 1459, 1230, 1157, 807, 742, 674 cm^{-1}. HRMS (ESI): Calcd. for $C_{24}H_{24}N_3$ [M+H]$^+$: 354.1965; found: 354.1969.

(11) 2-(2-methyl-1H-indol-3-yl)-7-(naphthalen-2-yl)-1,2,3,4-tetrahydro-1,8-naphthyr-idine (**3ka**)

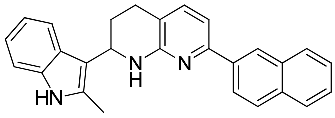

Yellow solid, (35.01 mg, 45% yield), m.p.: 193 - 194 °C; ^{1}H NMR (400 MHz, $CDCl_3$): δ 8.40 (s, 1H), 8.06 (d, J = 8.6 Hz, 1H), 7.87 (s, 1H), 7.85 - 7.74 (m, 3H), 7.64 (d, J = 7.7 Hz, 1H), 7.44 – 7.35 (m, 2H), 7.25 (d, J = 7.5 Hz, 1H), 7.11 – 7.07 (m, 2H), 7.03 (t, J = 7.4 Hz, 1H), 6.97 (t, J = 7.3 Hz, 1H), 5.13 (s, 1H), 4.75 (dd, J = 10.5, 2.4 Hz, 1H), 2.93 – 2.81 (m, 1H), 2.79 - 2.71(m, 1H), 2.39 - 2,27 (m, 1H), 2.14 (s, 3H), 2.00 – 1.91 (m, 1H). ^{13}C NMR (101 MHz, $CDCl_3$): δ 156.72, 153.85, 137.47, 137.08, 135.45, 133.72, 133.51, 131.94, 128.77, 128.23, 127.80, 127.01, 126.23, 125.82, 124.95, 121.21, 119.43, 119.37, 115.08, 112.57, 110.64, 110.23, 49.61, 28.70, 26.96, 11.97. IR (KBr): 3412, 3054, 2927, 2358, 1686, 1596, 1462, 1272, 1120, 1045, 808, 744, 473 cm^{-1}. HRMS (ESI): Calcd. for $C_{27}H_{24}N_3$ [M+H]$^+$: 390.1965; found: 390.1967.

(12) 2-(2-methyl-1H-indol-3-yl)-6-phenyl-1,2,3,4-tetrahydro-1,8-naphthyridine (**3la**)

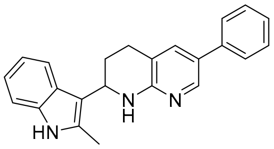

Yellow solid, (28.48 mg, 42% yield), ^{1}H NMR (400 MHz, DMSO) δ 10.92 (s, 1H), 7.61 (s, 1H), 7.56 - 7.50(m, 2H), 7.47 – 7.36 (m, 4H), 7.35 - 7.31 (d, *J* = 4.3 Hz, 2H), 7.26 (t, *J* = 6.3 Hz, 1H), 7.03 (t, *J* = 7.6 Hz, 1H), 6.91 (t, *J* = 7.4 Hz, 1H), 4.92 (d, *J* = 7.1 Hz, 1H), 3.00 - 2.88 (m, 1H), 2.79 (d, *J* = 16.1 Hz, 1H), 2.41 (s, 3H), 2.24 - 2.12 (m, 1H), 202 - 1.93(m, 1H). ^{13}C NMR (101 MHz, DMSO) δ 156.85, 143.72, 138.61, 135.74, 134.11, 132.59, 129.28, 127.38, 126.47, 125.58, 124.00, 120.46, 118.89, 118.75, 115.72, 112.38, 111.10, 48.97, 28.87, 26.80, 12.26. IR (KBr):3054, 295, 1670, 1608, 1490, 1298, 744, 696 cm^{-1}. HRMS (ESI): Calcd. For $C_{23}H_{22}N_3$ [M+H]$^+$: 340.1808；found: 340.1815.

(13) 2-(2-methyl-1H-indol-3-yl)-7-(1-methyl-1H-pyrrol-2-yl)-1,8-naphthyridine (**3ma**)

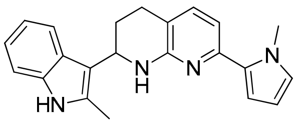

Yellow solid, (29.07 mg, 43% yield), m.p.: 343 - 344 °C; ^{1}H NMR (400 MHz, $CDCl_3$): δ 7.94 (s, 1H), 7.55 (d, *J* = 7.9 Hz, 1H), 7.16 -7.08 (m, 2H), 6.97 (t, *J* = 7.5 Hz, 1H), 6.89 (t, *J* = 7.4 Hz, 1H), 6.69 (d, *J* = 7.5 Hz, 1H), 6.52 (s, 1H), 6.37 (d, *J* = 1.7 Hz, 1H), 6.06 – 5.96 (m, 1H), 5.01 (s, 1H), 4.73 (dd, *J* = 10.7, 3.1 Hz, 1H), 3.76 (s, 3H), 2.84 – 2.61 (m, 2H), 2.32 – 2.10 (m, 4H), 1.92 (dd, *J* = 7.8, 3.0 Hz, 1H). ^{13}C NMR (101 MHz, $CDCl_3$): δ 155.56, 148.88, 136.76, 135.38, 132.83, 131.80, 126.88, 125.32, 121.18, 119.38, 119.24, 112.92, 112.66, 111.09, 110.50, 109.88, 107.39, 49.53, 36.53, 28.71, 26.77, 12.01. IR (KBr): 2929, 2357, 1686, 1593, 1461, 1374, 1293, 1117, 1066, 735 cm^{-1}.HRMS (ESI): Calcd. for $C_{22}H_{23}N_4$ [M+H]$^+$: 343.1920; found: 343.1922.

(14) 2-(2-methyl-1H-indol-3-yl)-7-(thiophen-2-yl)-1,2,3,4-tetrahydro-1,8-naphthyridine (3na)

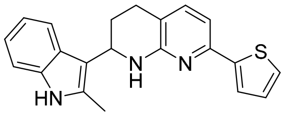

Yellow oily liquid, (41.40 mg, 60% yield), ^{1}H NMR (400 MHz, $CDCl_3$) δ 8.18 (s, 1H), 7.83 (d, *J* = 3.1 Hz, 1H), 7.65 (d, *J* = 7.9 Hz, 1H), 7.44 (d, *J* = 7.4 Hz, 1H), 7.35 – 7.19 (m, 4H), 7.10 (t, *J* = 7.5 Hz, 1H), 7.02 (t, *J* = 7.5 Hz, 1H), 5.16 (s, 1H), 4.82 (dd, *J* = 10.7, 3.0 Hz, 1H), 3.03 – 2.81 (m, 2H), 2.44 – 2.34 (m, 4H), 2.04 (dd, *J* = 8.9, 4.1 Hz, 1H). ^{13}C NMR (101 MHz, $CDCl_3$) δ 170.51, 156.24, 147.63, 143.54, 136.82, 135.42, 131.86, 126.83, 121.23, 120.25, 119.41, 119.26, 117.78, 112.34, 110.52, 108.81, 49.41, 28.24, 27.10, 11.99. IR (KBr):3405, 3063, 2928, 2848, 1590, 1462, 1110, 915, 873, 737 cm^{-1}. HRMS (ESI): Calcd. For $C_{21}H_{20}N_3S$ [M+H]$^{+}$:346.1332; found:346.1337.

(15) 2,3-dimethyl-6-(2-methyl-1H-indol-3-yl)-5,6,7,8-tetrahydropyrido[2,3-b]pyr Azine (**3oa**)

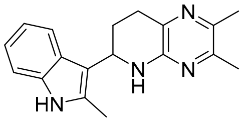

Yellow oily liquid, (32.70 mg, 56% yield), ^{1}H NMR (400 MHz, $CDCl_3$) δ 8.31 (s, 1H), 7.62 (d, *J* = 7.9 Hz, 1H), 7.25 (s, 1H), 7.09 (t, *J* = 7.5 Hz, 1H), 7.01 (t, J = 7.5 Hz, 1H), 4.90 (s, 1H), 4.82 (dd, *J* = 10.8, 2.9 Hz, 1H), 3.08 – 2.93 (m, 2H), 2.51 – 2.34 (m, 8H), 2.31 (s, 3H). ^{13}C NMR (101 MHz, $CDCl_3$) δ 150.38, 146.81, 138.41, 135.31, 134.98, 131.71, 126.78, 121.23, 119.41, 119.06, 112.05, 110.41, 49.36, 29.87, 28.70, 21.18, 20.46, 12.08. IR (KBr): 3262, 2927, 1687, 1560, 1433, 1330, 978, 913, 739 cm^{-1}. HRMS (ESI): Calcd. For $C_{18}H_{21}N_4$ [M+H]$^{+}$: 293.1761; found:293.1768.

(16) 2-(2-methyl-1H-indol-3-yl)-1,2,3,4,6,7,8,9-octahydropyrido[2,3-b]quinoxaline (**3pa**)

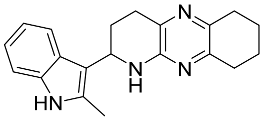

Yellow oily liquid, (33.07 mg, 52% yield), ^{1}H NMR (400 MHz, $CDCl_3$) δ 8.15 (s, 1H), 7.63 (d, J = 7.9 Hz, 1H), 7.26 (d, J = 6.3 Hz, 1H), 7.10 (t, J = 7.5 Hz, 1H), 7.05 - 6.99 (m，1H), 4.92 (s, 1H), 4.85 (dd, J = 10.8, 2.8 Hz, 1H), 3.12 - 2.97 (m, 2H), 2.84 - 2.77 (m, 2H), 2.74 - 2.66 (m, 2H), 2.50 – 2.40 (m, 4H), 2.17 – 2.05 (m, 2H), 1.91 – 1.84 (m, 3H). ^{13}C NMR (101 MHz, $CDCl_3$) δ 150.30, 147.32, 139.32, 136.08, 135.27, 131.65, 126.76, 121.29, 119.47, 119.06, 112.07, 110.38, 49.42, 31.36, 30.75, 30.17, 28.74, 23.24, 22.91, 12.13. IR (KBr): 3257, 2933, 1688, 1562, 1432, 739 cm^{-1}. HRMS (ESI): Calcd. For $C_{20}H_{23}N_4$ $[M+H]^+$: 319.1917； found:319.1925.

(17) 2-(5-methoxy-1H-indol-3-yl)-7-(4-(trifluoromethyl)phenyl)-1,2,3,4-tetrahydro-1,8-naphthyridine (**3ab**)

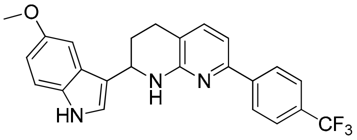

Yellow solid, (56.68 mg, 67% yield), m.p.: 173 - 174 °C; ^{1}H NMR (400 MHz, $CDCl_3$): δ 8.35 (s, 1H), 8.07 (d, *J* = 8.1 Hz, 2H), 7.70 (d, *J* = 8.2 Hz, 2H), 7.35 (d, *J* = 7.5 Hz, 1H), 7.21 (d, *J* = 8.8 Hz, 1H), 7.13 (s, 2H), 7.06 (d, *J* = 7.5 Hz, 1H), 6.91 (dd, *J* = 8.8, 2.2 Hz, 1H), 5.38 (s, 1H), 4.98 (dd, *J* = 8.2, 2.9 Hz, 1H), 3.87 (s, 3H), 2.99 - 2.89 (m, 1H), 2.87 - 2.78 (m, 1H), 2.32 - 2.18 (m, 2H). ^{13}C NMR (101 MHz, $CDCl_3$) δ 156.31 (s), 154.04 (s), 152.40 (s), 143.28 (s), 136.98 (s), 131.78 (s), 130.00 (q, *J* = 64.6, 32.3 Hz), 126.87 (s), 125.86 (s), 125.47 (q, *J* = 7.4, 3.6 Hz), 123.03 (s), 122.38 (s), 118.41 (s), 116.02 (s), 112.41 (s), 112.19 (s), 110.29 (s), 100.99 (s), 56.01 (s), 49.12 (s), 28.47 (s), 25.57 (s). IR (KBr): 3414, 2929, 2358, 1588, 1469, 1215, 1166, 804, 661 cm^{-1}. HRMS (ESI): Calcd. for $C_{24}H_{21}F_3N_3O$ [M+H]: 424.1631; found: 424.1634.

(18) 2-(6-methyl-1H-indol-3-yl)-7-(4-(trifluoromethyl)phenyl)-1,2,3,4-tetrahydro-1,8-naphthyridine (**3ac**)

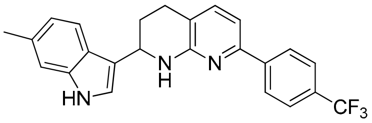

Yellow solid, (50.47 mg, 62% yield), m.p.: 173 - 174 °C; ^{1}H NMR (400 MHz, DMSO): δ 10.75 (s, 1H), 8.21 (d, *J* = 8.3 Hz, 2H), 7.78 (d, *J* = 8.3 Hz, 2H), 7.48 (d, *J* = 8.1 Hz, 1H), 7.34 (d, *J* = 7.4 Hz, 1H), 7.15 (d, *J* = 7.6 Hz, 2H), 7.10 (d, *J* = 2.1 Hz, 1H), 6.82 (d, *J* = 8.0 Hz, 1H), 6.73 (s, 1H), 4.93 (s, 1H), 2.85 - 2.75 (m, 1H), 2.67 - 2.58 (m, 1H), 2.39 (s, 3H), 2.08 (d, *J* = 5.2 Hz, 2H). ^{13}C NMR (101 MHz, DMSO) δ 156.66 (s), 151.03 (s), 143.71 (s), 137.58 (s), 137.01 (s), 130.52 (s), 128.55 (s), 127.14 (s), 125.76 (q, *J* = 3.7 Hz), 123.59 (s), 122.21 (s), 120.67 (s), 118.89 (s), 117.99 (s), 116.23 (s), 111.87 (s), 109.36 (s), 48.66 (s), 28.39 (s), 24.89 (s), 21.84 (s). IR (KBr): 3428, 2358, 1606, 1462, 1322, 807, 741, 674 cm^{-1}. HRMS (ESI): Calcd. for $C_{24}H_{21}F_3N_3$ [M+H]$^+$: 408.1682; found: 408. 1686.

(19) 2-(7-methyl-1H-indol-3-yl)-7-(4-(trifluoromethyl)phenyl)-1,2,3,4-tetrahydro-1,8-naphthyridine (**3ad**)

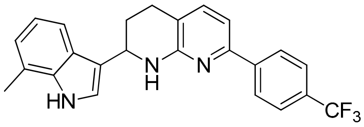

Yellow solid, (49.65 mg, 61% yield), m.p.: 175 - 176 °C; ^{1}H NMR (400 MHz, $CDCl_3$): δ 8.23 (s, 1H), 8.11 (d, *J* = 8.2 Hz, 2H), 7.72 (d, *J* = 8.3 Hz, 2H), 7.60 (d, *J* = 7.6 Hz, 1H), 7.36 (d, *J* = 7.5 Hz, 1H), 7.16 - 7.07 (m, 4H), 5.43 (s, 1H), 5.02 (dd, *J* = 8.0, 3.3 Hz, 1H), 2.97 – 2.89 (m, 1H), 2.87 - 2.78 (m, 1H), 2.49 (s, 3H), 2.34 – 2.22 (m, 2H). ^{13}C NMR (101 MHz, $CDCl_3$): ^{13}C NMR (101 MHz, $CDCl_3$) δ 156.36 (s), 152.43 (s), 136.97 (s), 136.29 (s), 129.99 (q, *J* = 32.4 Hz), 126.90 (s), 125.47 (q, *J* =3.6 Hz), 125.00 (s), 122.92 (s), 121.30 (s), 120.78 (s), 119.94 (s), 119.33 (s), 116.77 (s), 116.03 (s), 110.28 (s), 49.30 (s), 28.66 (s), 25.53 (s), 16.60 (s). IR (KBr): 3428, 2358, 1606, 1462, 1322, 807, 741, 674cm^{-1}. HRMS (ESI): Calcd. for $C_{24}H_{21}F_3N_3$ [M+H]$^+$:408.1682; found: 408.1687.

(20) 2-(7-(5-methoxy-1H-indol-3-yl)-5,6,7,8-tetrahydro-1,8-naphthyridin-2-yl)phenol (**3hb**)

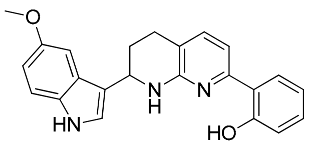

Yellow solid,(46.75 mg, 63% yield), m.p.: 206 - 207 °C; ^{1}H NMR (400 MHz, DMSO-d_6): δ 14.30 (s, 1H), 10.80 (s, 1H), 7.83 (d, J = 8.0 Hz, 1H), 7.56 (s, 1H), 7.38 (d, J = 7.7 Hz, 1H), 7.29 (d, J = 8.8 Hz, 1H), 7.22 - 7.15 (m, 3H), 7.10 (d, J = 1.9 Hz, 1H), 6.86 - 6.80 (m, 2H), 6.76 (dd, J = 8.8, 2.2 Hz, 1H), 4.93 (t, J = 4.6 Hz, 1H), 3.73 (s, 3H), 2.87 - 2.74 (m, 1H), 2.70 - 2.60 (m, 1H), 2.16 - 2.04 (m 2H). ^{13}C NMR (101 MHz, DMSO-d_6): δ 159.38, 154.18, 153.48, 153.34, 137.82, 132.21, 130.57, 126.75, 126.13, 123.71, 119.91, 118.76, 118.20, 117.49, 114.59, 112.72, 111.65, 106.96, 101.27, 55.85, 48.42, 28.13, 24.94. IR (KBr): 3057, 2358, 1593, 1470, 1271, 1213, 746 cm^{-1}. HRMS (ESI): Calcd. for $C_{23}H_{22}N_3O_2$ [M+H]$^+$:372.1707; found：372.1710.

(21) 2-(7-(6-methyl-1H-indol-3-yl)-5,6,7,8-tetrahydro-1,8-naphthyridin-2-yl)phenol (**3hc**)

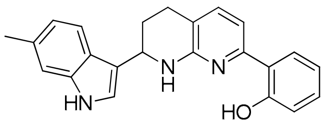

Yellow solid, (43.31mg, 61% yield), m.p.: 203 - 204 °C; ^{1}H NMR (400 MHz, DMSO-d_6): δ 14.29 (s, 1H), 10.78 (s, 1H), 7.82 (d, J = 7.9 Hz, 1H), 7.54 (s, 1H), 7.48 (d, J = 8.1 Hz, 1H), 7.37 (d, J = 7.7 Hz, 1H), 7.23 - 7.15 (m, 3H), 7.13 (s, 1H), 6.88 - 6.78 (m, 3H), 4.91 (s, 1H), 2.86 - 2.71 (m, 1H), 2.66 - 2.56 (m, 1H), 2.38 (s, 3H), 2.08 (d, J = 5.3 Hz, 2H). ^{13}C NMR (101 MHz, DMSO-d_6): δ 159.38, 154.14, 153.31, 137.80, 137.59, 130.56, 126.75, 123.70, 122.38, 120.72, 119.91, 119.03, 118.76, 118.19, 117.53, 114.56, 111.89, 106.96, 48.64, 28.33, 24.99, 21.85. IR (KBr): 2925, 1595, 1514, 1467, 1278, 804, 748 cm^{-1}. HRMS (ESI): Calcd. for $C_{23}H_{22}N_3O$ [M+H]$^+$: 356.1757; found: 356.1761.

(22) 7-(7-methyl-1H-indol-3-yl)-2-phenyl-5,6,7,8-tetrahydro-1,8-naphthyridine-3-carbonitrile (**3bd**)

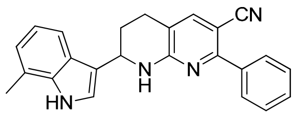

Yellow solid, (51.69mg, 71% yield), m.p.: 172 - 173 °C; ^{1}H NMR (400 MHz, $CDCl_3$) δ 8.21 (s, 1H), 7.87 (dd, J = 7.6, 1.8 Hz, 2H), 7.53 – 7.41 (m, 5H), 7.16 - 7.05(m, 3H), 5.96 (s, 1H), 5.10 - 4.97 (m, 1H), 2.89 - 2.69 (m, 2H), 2.51 (s, 3H), 2.32 - 2.15 (m, 2H). ^{13}C NMR (101 MHz, $CDCl_3$) δ 159.88, 157.25, 139.84, 137.87, 136.30, 129.57, 128.71, 128.70, 128.55, 128.47, 124.62, 123.11, 121.36, 120.86, 120.13, 119.84, 118.03, 116.52, 114.57, 94.30, 49.46, 27.67, 24.82, 16.62. IR (KBr): 3057, 2358, 2212, 1712, 1601, 1437, 919, 746, 696 cm^{-1}. HRMS (ESI): Calcd. for $C_{20}H_{17}N_2O_2$ $[M+H]^+$: 365.1761; found: 365.1765.

(23) 5-nitro-2-(1H-pyrrol-3-yl)quinoline (**3kb**)

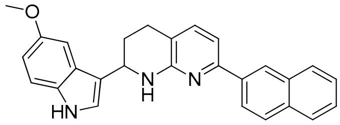

Yellow solid, (43.74 mg, 54% yield), m.p.: 205 - 206 °C; ^{1}H NMR (400 MHz, DMSO) δ 10.77 (s, 1H), 8.55 (s, 1H), 8.23 - 8.17 (m, 1H), 8.00 - 7.90 (m, 3H), 7.57 - 7.50 (m, 2H), 7.35 (d, J = 7.4 Hz, 1H), 7.29 (d, J = 8.8 Hz, 1H), 7.24 - 7.17 (m, 2H), 7.12 (d, J = 2.1 Hz, 1H), 6.77 (dd, J = 8.8, 2.2 Hz, 1H), 6.65 (s, 1H), 4.97 (s, 1H), 3.74 (s, 3H), 2.88 - 2.75 (m, 1H), 2.72 - 2.60 (m, 1H), 2.24 - 1.99 (m, 2H). ^{13}C NMR (101 MHz, DMSO-d_6): δ 156.66, 153.47, 152.64, 137.34, 136.99, 133.57, 133.34, 132.26, 128.82, 128.23, 127.96, 126.70, 126.59, 126.07, 125.30, 124.93, 123.53, 118.10, 115.15, 112.71, 111.61, 109.14, 101.22, 55.88, 48.54, 28.41, 24.88. IR (KBr): 3054, 2927, 2358, 1686, 1595, 1462, 808, 744 cm^{-1}. HRMS (ESI): Calcd. for $C_{27}H_{24}N_3O$ $[M+H]^+$: 406.1914; found: 406.1918.

(24) 2-(5-(benzyloxy)-1H-indol-3-yl)-7-(4-(trifluoromethyl)phenyl)-1,2,3,4-tetrahydro-1,8-naphthyridine (**3ae**)

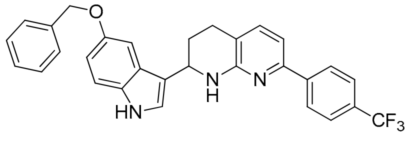

Yellow solid, (30.87 mg, 63% yield), m.p.: 207 - 208 °C; ^{1}H NMR (400 MHz, $CDCl_3$): δ 8.22 (s, 1H), 8.01 (d, *J* = 8.2 Hz, 2H), 7.63 (d, *J* = 8.2 Hz, 2H), 7.41 (d, *J* = 7.3 Hz, 2H), 7.33 (t, *J* = 7.3 Hz, 2H), 7.30 - 7.24 (t, *J* = 7.0 Hz, 2H), 7.18 - 7.10 (m, 2H), 7.01 (dd, *J* = 9.8, 4.8 Hz, 2H), 6.92 (dd, *J* = 8.8, 2.0 Hz, 1H), 5.28 (s, 1H), 5.04 (s, 2H), 4.87 (dd, *J* = 8.3, 3.0 Hz, 1H), 2.91 - 2.80 (m 1H), 2.73 (m, 1H), 2.20 - 2.06 (m, 2H). ^{13}C NMR (101 MHz, $CDCl_3$) ^{13}C NMR (101 MHz, $CDCl_3$) δ 156.34 (s), 153.21 (s), 152.43 (s), 143.35 (s), 137.61 (s), 136.95 (s), 132.02 (s), 130.02 (q, *J* = 32.3 Hz), 128.55 (s), 127.86 (s), 127.69 (s), 126.88 (s), 125.87 (s), 125.77 (s), 125.47 (q, *J* = 3.7 Hz), 123.07 (s), 122.44 (s), 118.44 (s), 116.03 (s), 113.08 (s), 112.18 (s), 110.29 (s), 102.87 (s), 71.13 (s), 49.21 (s), 28.51 (s), 25.60 (s). IR (KBr): 3253, 2919, 2849, 1615, 1554, 1443, 1167, 1128, 943, 738, 674 cm^{-1}. HRMS (ESI): Calcd. for $C_{30}H_{25}F_3N_3O$ $[M+H]^+$: 246.1022; found: 246.1026.

(25) 2-(7-(5-bromo-2-methyl-1H-indol-3-yl)-5,6,7,8-tetrahydro-1,8-naphthyridin-2-y-l)phenol (**3hf**)

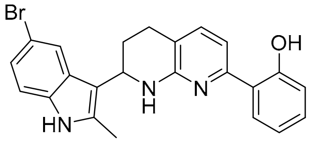

Yellow solid, (44.17 mg, 51% yield), ^{1}H NMR (400 MHz, $CDCl_3$) δ 8.21 (s, 1H), 7.74 (d, *J* = 9.8 Hz, 2H), 7.38 (d, *J* = 7.6 Hz, 1H), 7.23 (t, *J* = 7.4 Hz, 1H), 7.17 – 7.06 (m, 3H), 6.96 - 6.85 (dd, *J* = 17.3, 8.0 Hz, 2H), 5.11 (s, 1H), 4.76 (d, *J* = 10.5 Hz, 1H), 3.04 – 2.79 (m, 2H), 2.42 - 2.30 (m, 4H), 2.03 (d, *J* = 12.4 Hz, 1H). ^{13}C NMR (101 MHz, DMSO) δ 159.35, 154.47, 153.34, 137.87, 134.58, 134.44, 130.57, 129.16, 126.76, 122.83, 121.11, 119.91, 118.75, 118.20, 114.69, 113.01, 112.07, 111.44, 107.09, 48.53, 28.54, 26.27, 12.17. IR (KBr):3317, 2923, 1731, 1465, 1274, 907, 859, 718 cm^{-1}. HRMS (ESI): Calcd. For $C_{23}H_{21}BrN_3O$ $[M+H]^+$: 434.0863; found: 434.0873.

(26) 2-(7-(5-fluoro-2-methyl-1H-indol-3-yl)-5,6,7,8-tetrahydro-1,8-naphthyridin-2-yl)phenol (**3hg**)

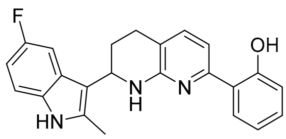

Yellow solid, (46.25 mg, 62% yield), ^{1}H NMR (400 MHz, $CDCl_3$) δ 8.11 (s, 1H), 7.68 (d, *J* = 7.9 Hz, 1H), 7.30 (d, *J* = 7.8 Hz, 1H), 7.21 – 7.15 (m, 2H), 7.09 - 7.03 (m, 2H), 6.89 – 6.80 (m, 2H), 6.73 (td, *J* = 9.0, 2.2 Hz, 1H), 5.04 (s, 1H), 4.69 (dd, *J* = 10.7, 3.0 Hz, 1H), 2.93 – 2.81 (m, 1H), 2.80 - 2.73(m, 1H), 2.29 – 2.18 (m, 4H), 2.00 – 1.92 (m, 1H). ^{13}C NMR (101 MHz, $CDCl_3$) ^{13}C NMR (101 MHz, $CDCl_3$) δ 159.18 (s), 157.55 (d, *J* = 233.8 Hz), 153.69 (s), 153.37 (s), 137.68 (s), 133.97 (s), 131.86 (s), 130.37 (s), 126.99 (d, *J* = 9.9 Hz), 126.13 (s), 119.69 (s), 118.72 (s), 118.11 (s), 114.36 (s), 112.21 (d, *J* = 4.5 Hz), 111.09 (d, *J* = 9.7 Hz), 109.20 (d, *J* = 26.0 Hz), 107.93 (s), 104.03 (d, *J* = 24.0 Hz), 49.31 (s), 28.19 (s), 26.60 (s), 11.96 (s). IR (KBr): 3413, 2927, 1595, 1474, 1280, 930, 801, 749, 605 cm^{-1}. HRMS (ESI): Calcd. For $C_{23}H_{21}FN_3O$ $[M+H]^+$: 434.0863；374.1671, found: 374.1675

NMR spectra of the obtained compounds

^{1}H-NMR spectrum of 3aa

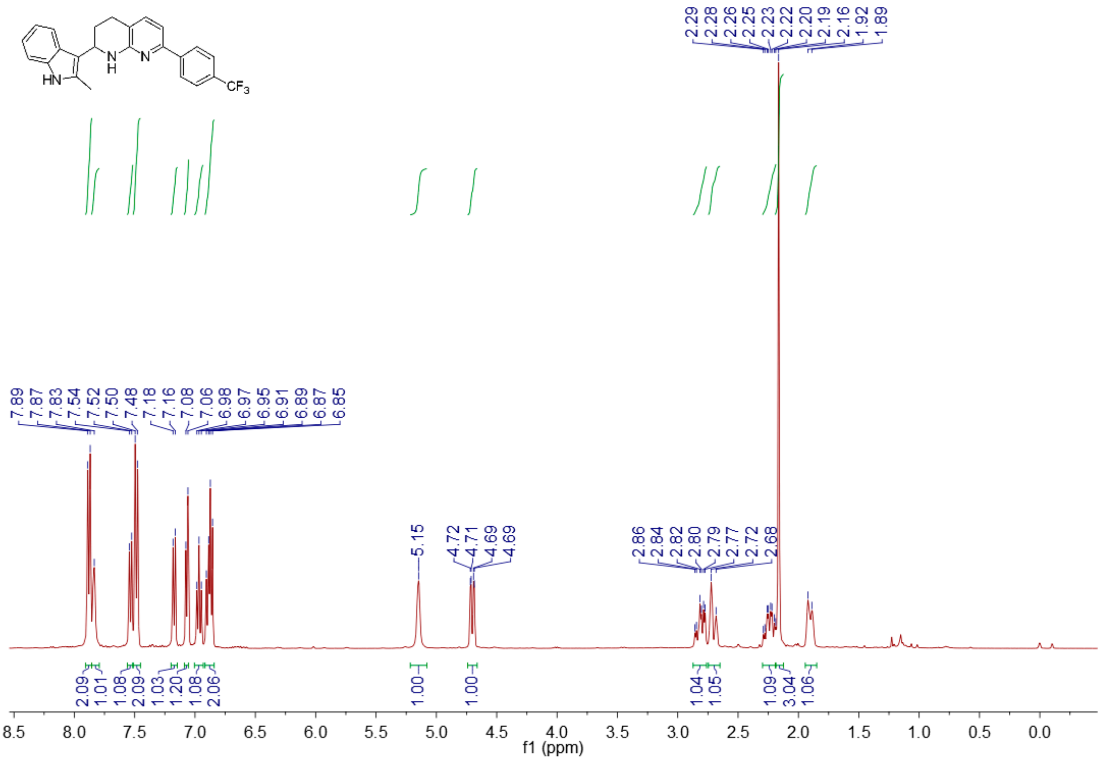

^{13}C-NMR spectrum of 3aa

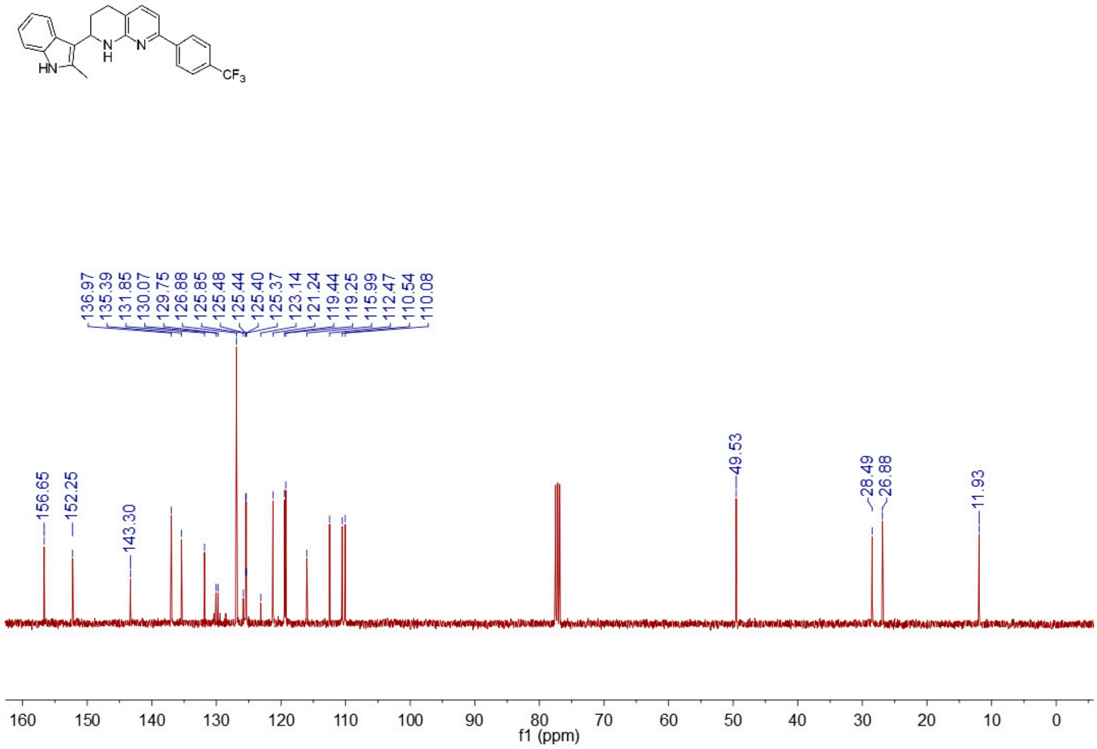

^{1}H-NMR spectrum of 3ba

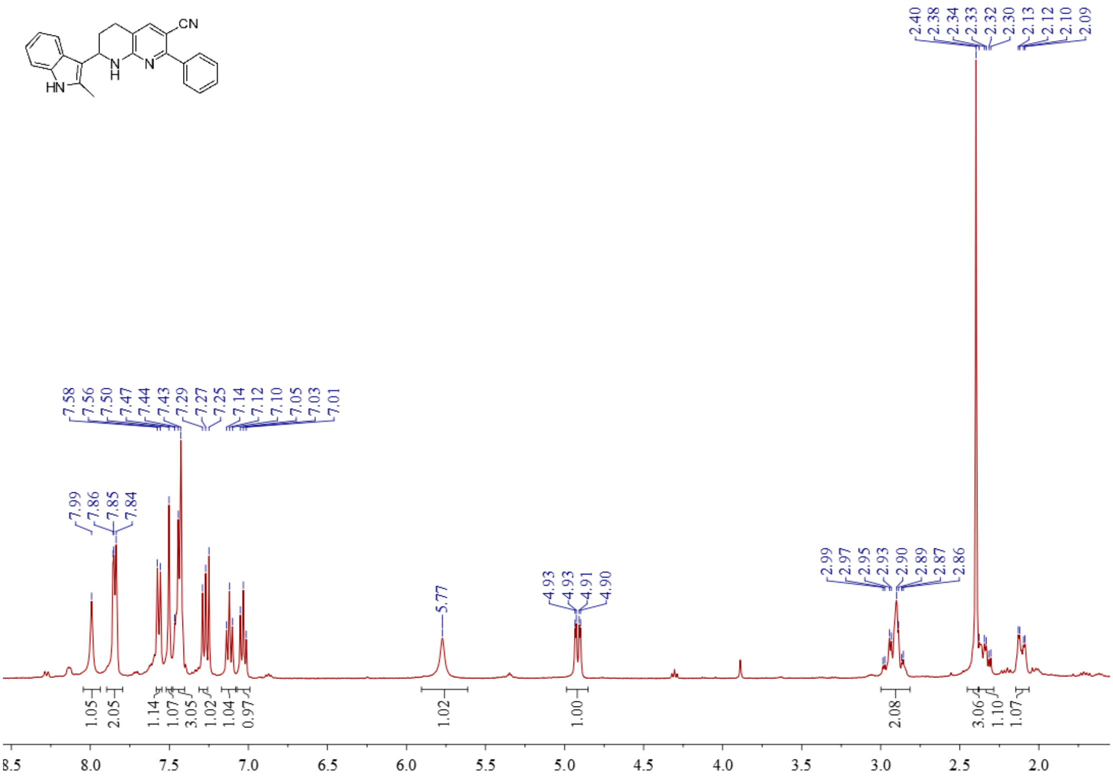

^{13}C-NMR spectrum of 3ba

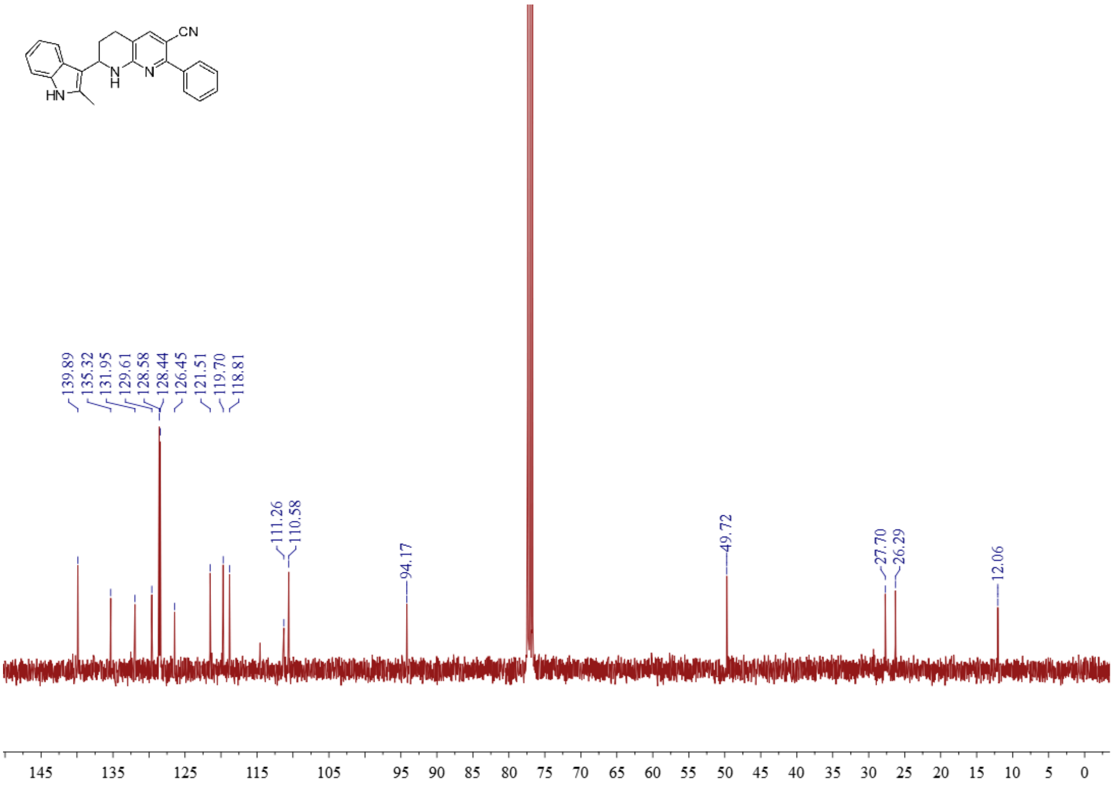

^{1}H-NMR spectrum of 3ca

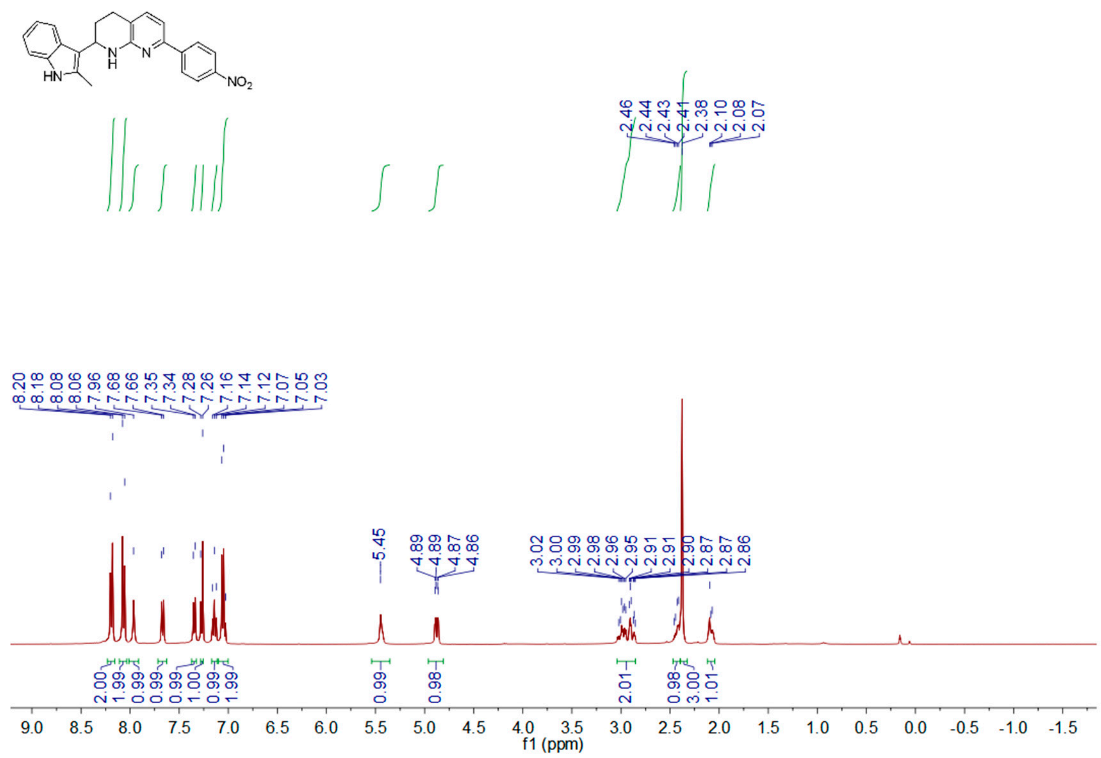

^{13}C-NMR spectrum of 3ca

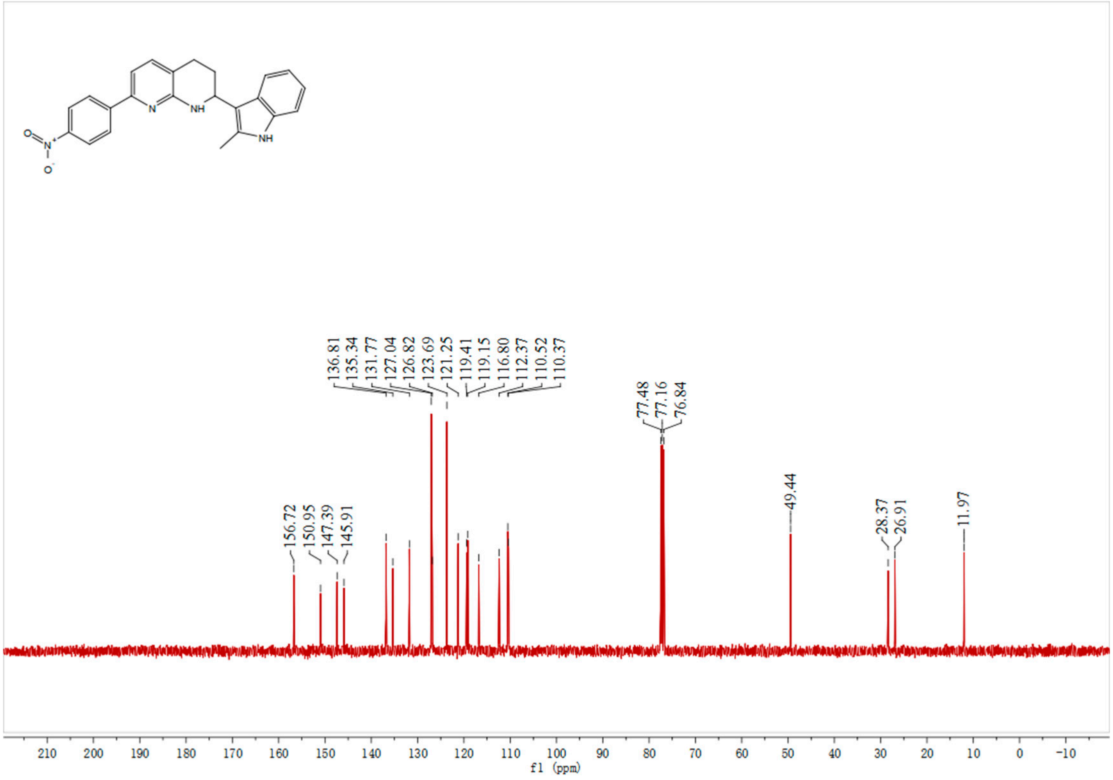

---

^{1}H-NMR spectrum of 3da

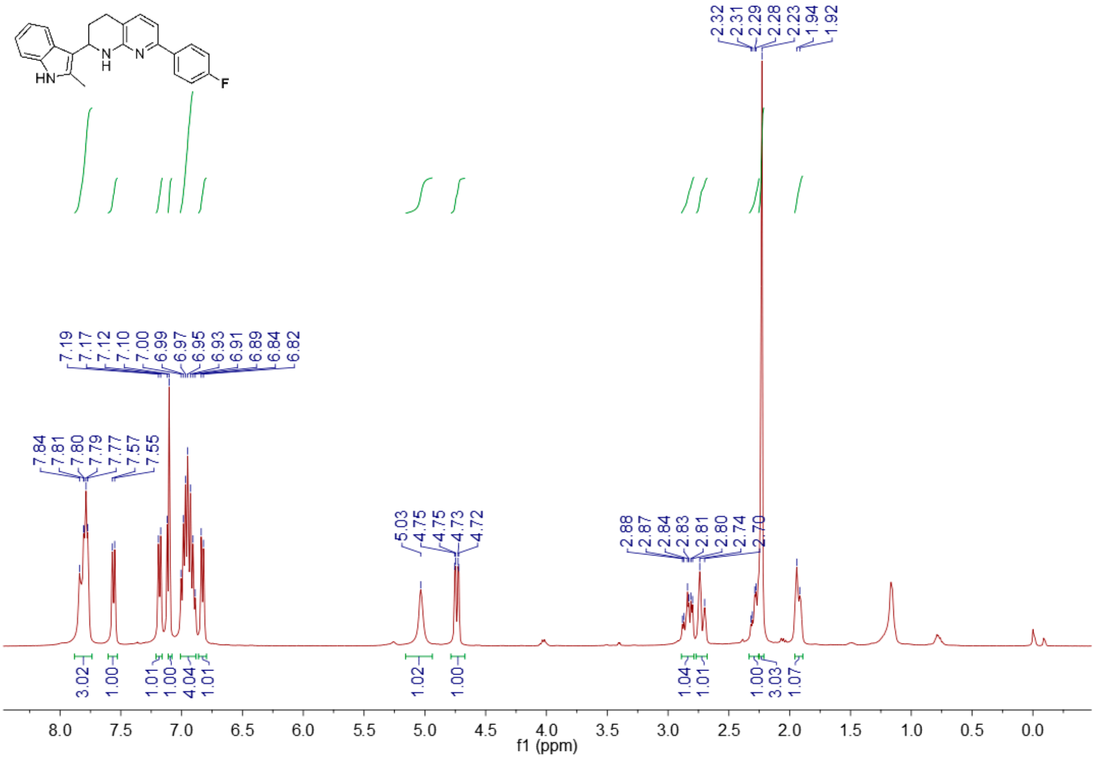

^{13}C-NMR spectrum of 3da

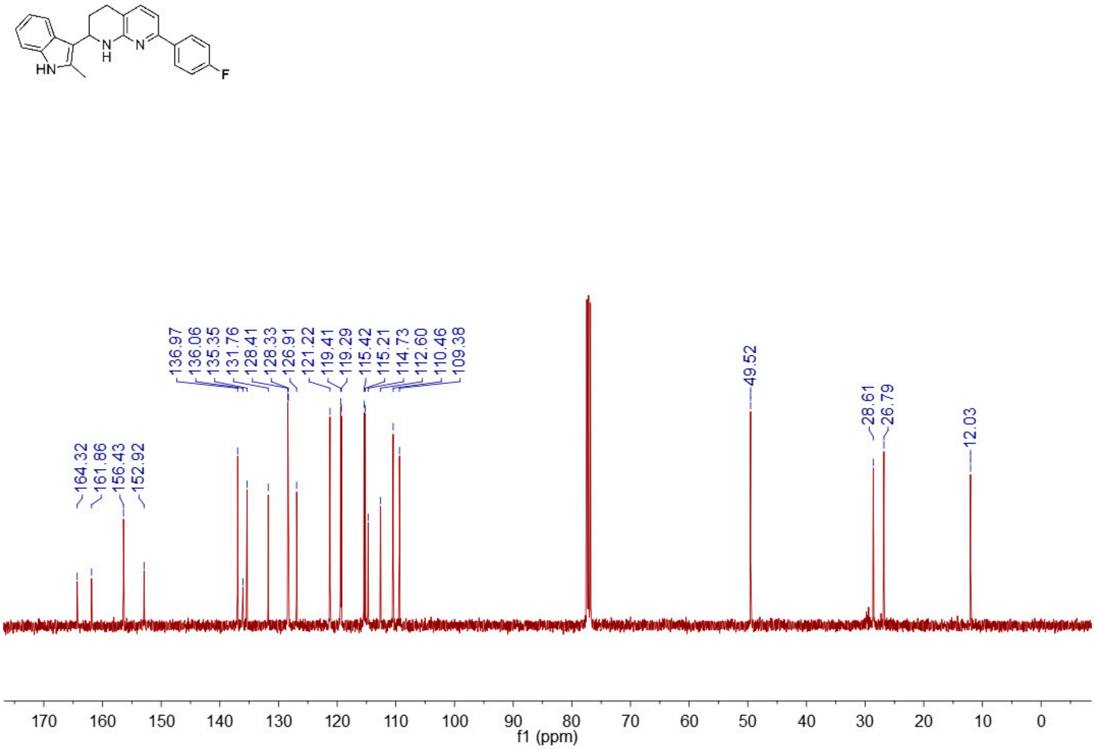

^{1}H-NMR spectrum of 3ea

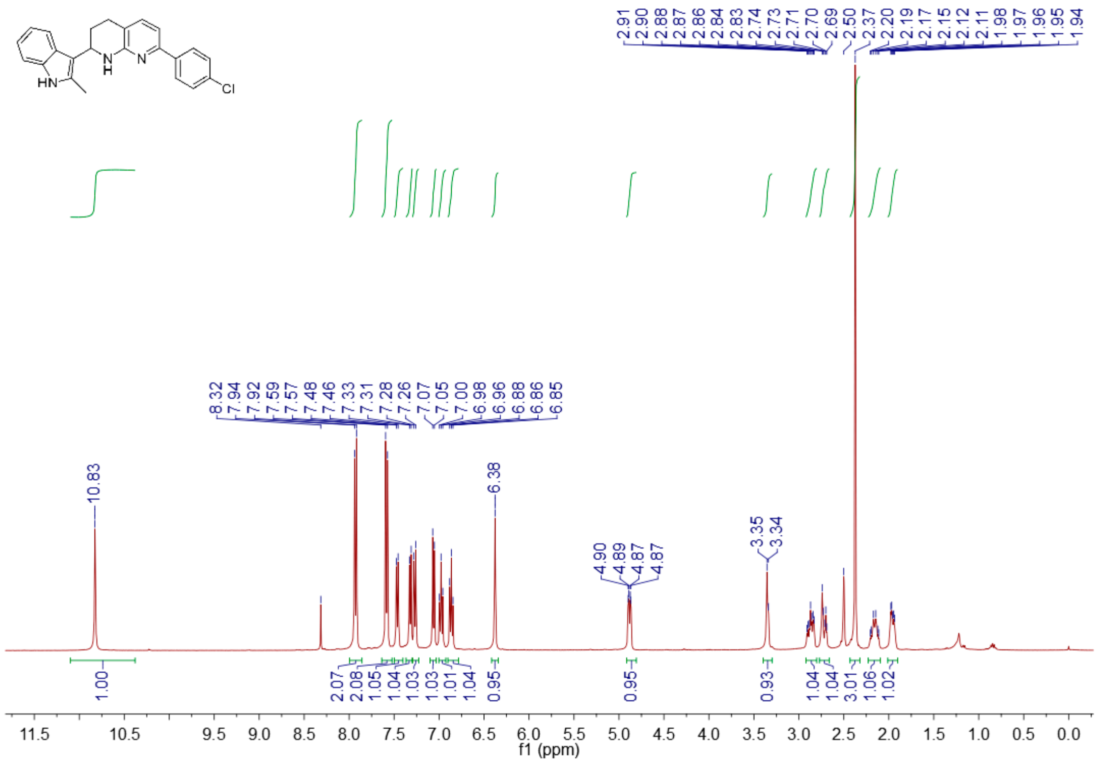

^{13}C-NMR spectrum of 3ea

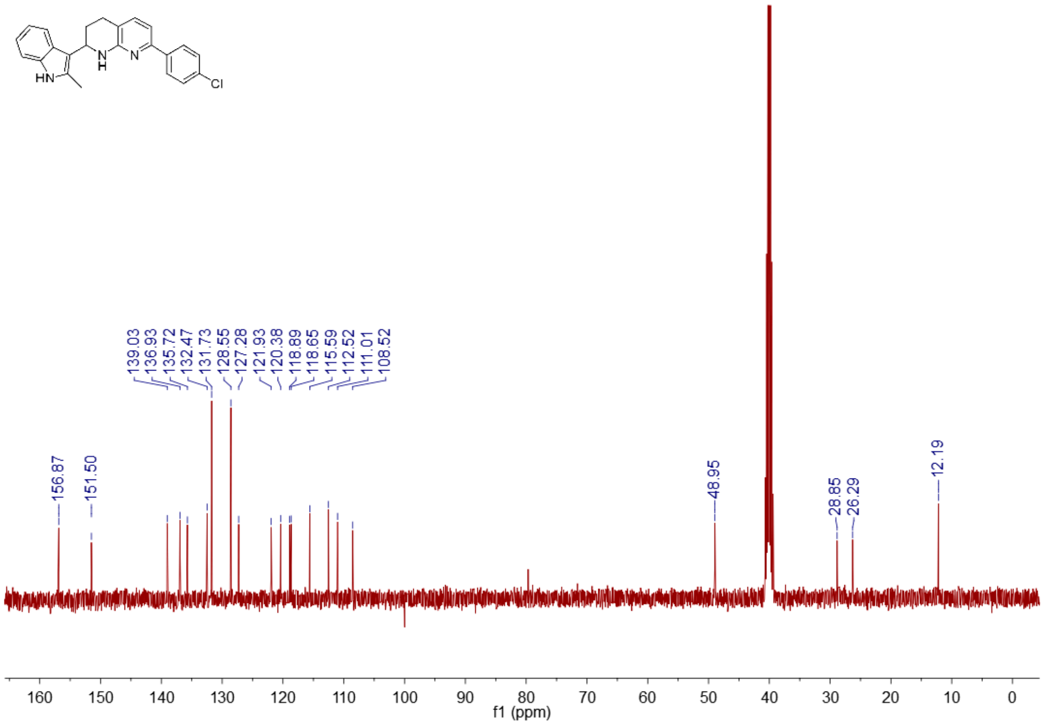

^{1}H-NMR spectrum of 3fa

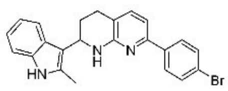

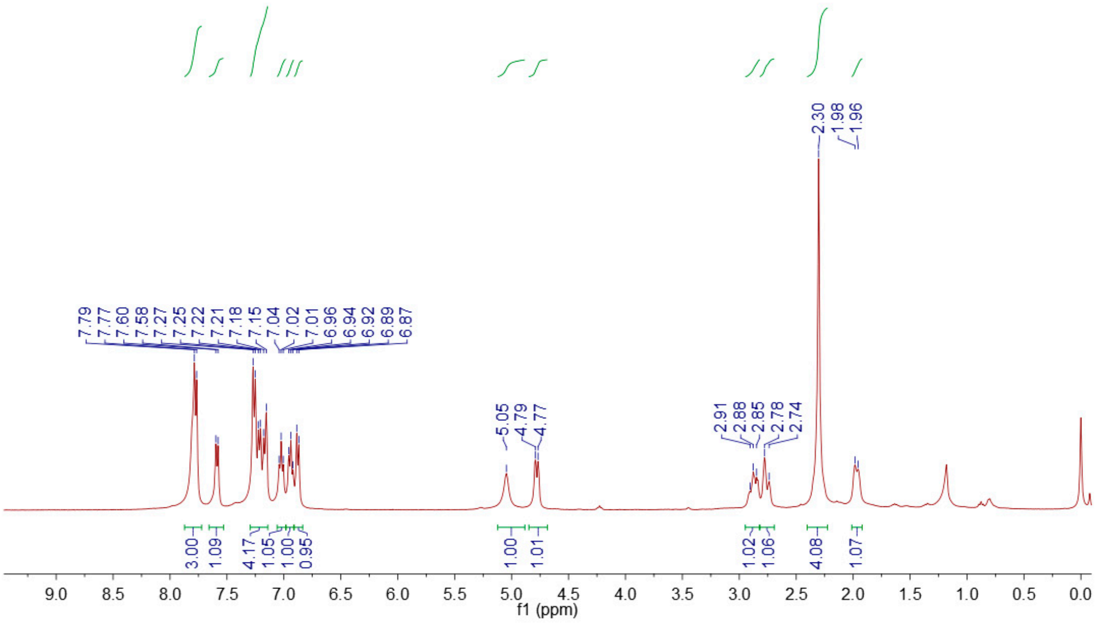

^{13}C-NMR spectrum of 3fa

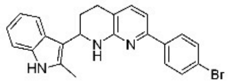

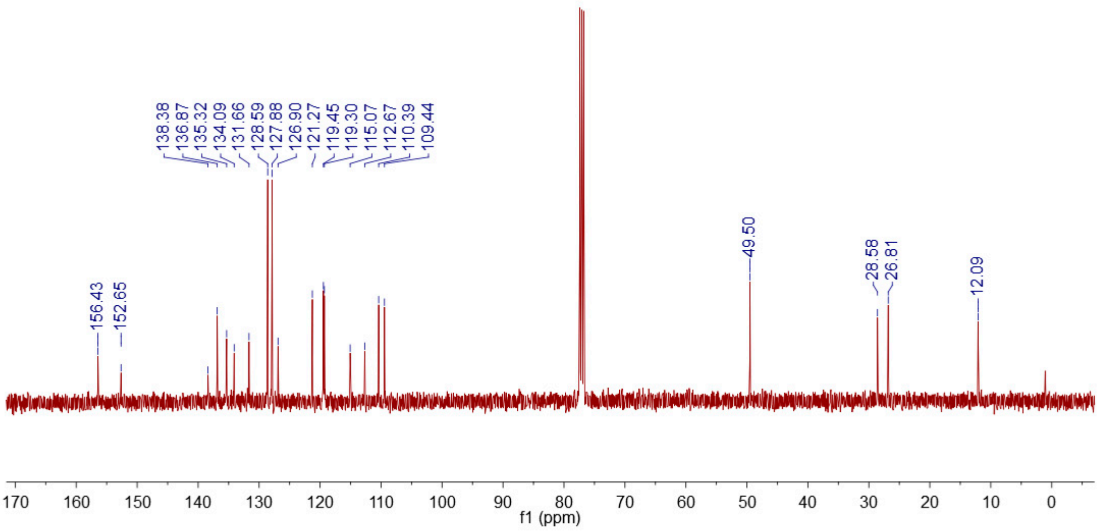

^{1}H-NMR spectrum of 3ga

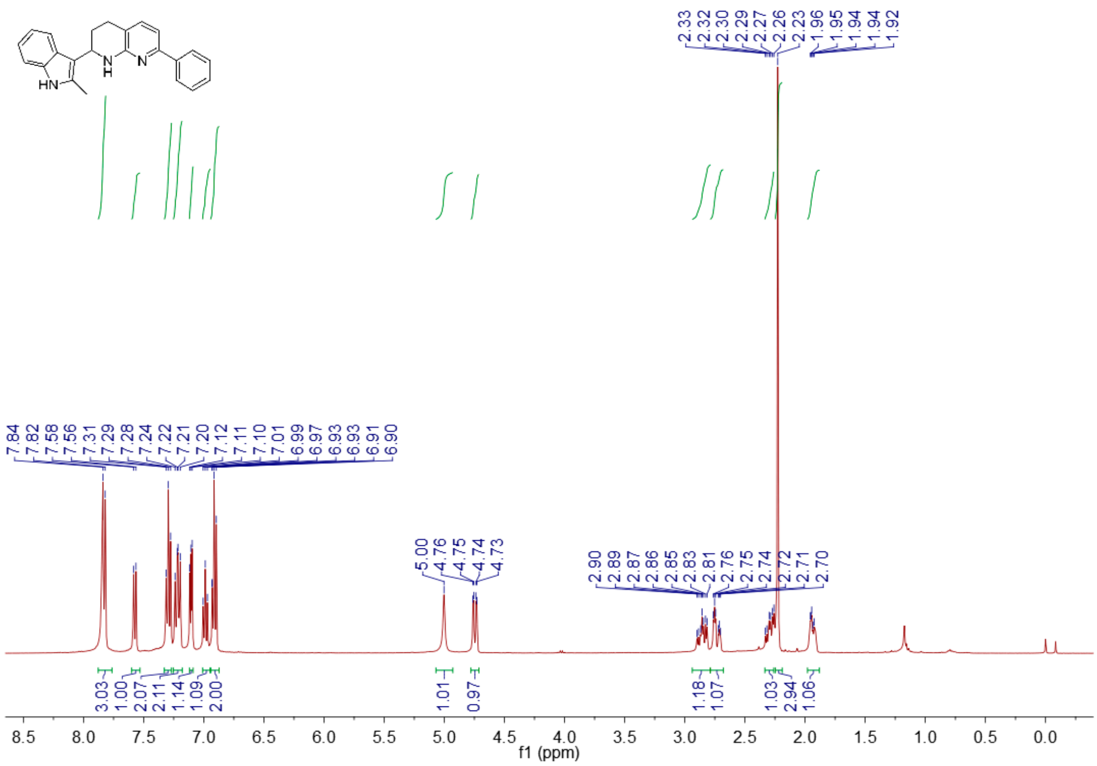

^{13}C-NMR spectrum of 3ga

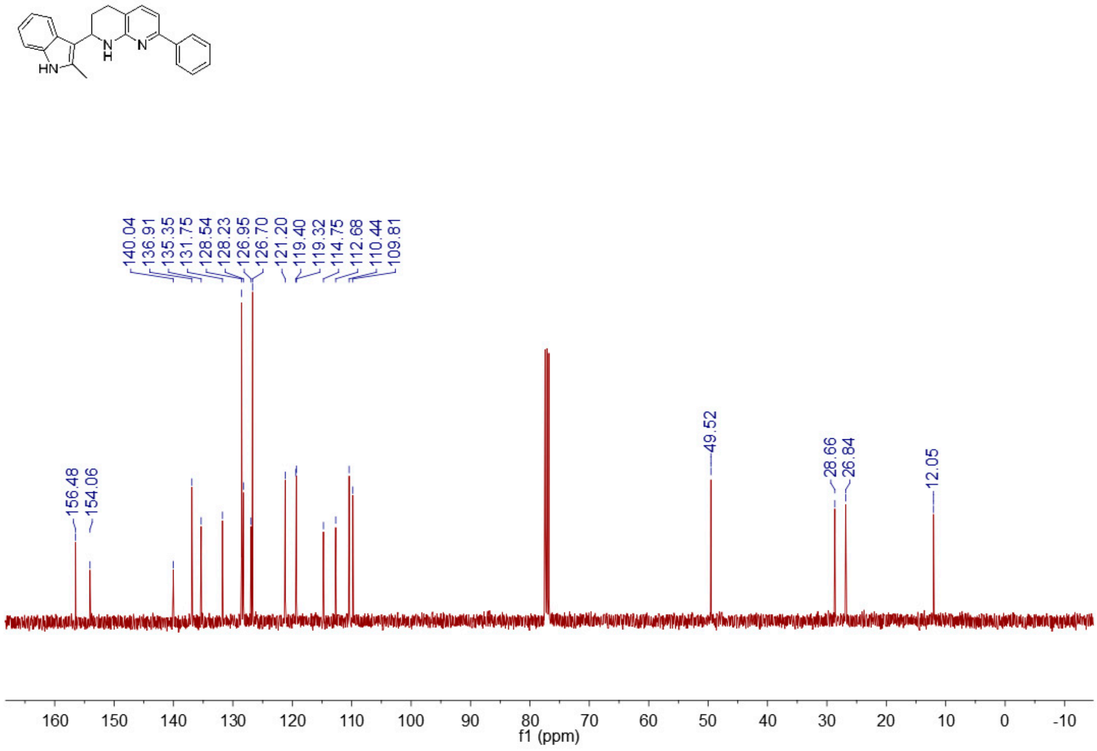

^{1}H-NMR spectrum of 3ha

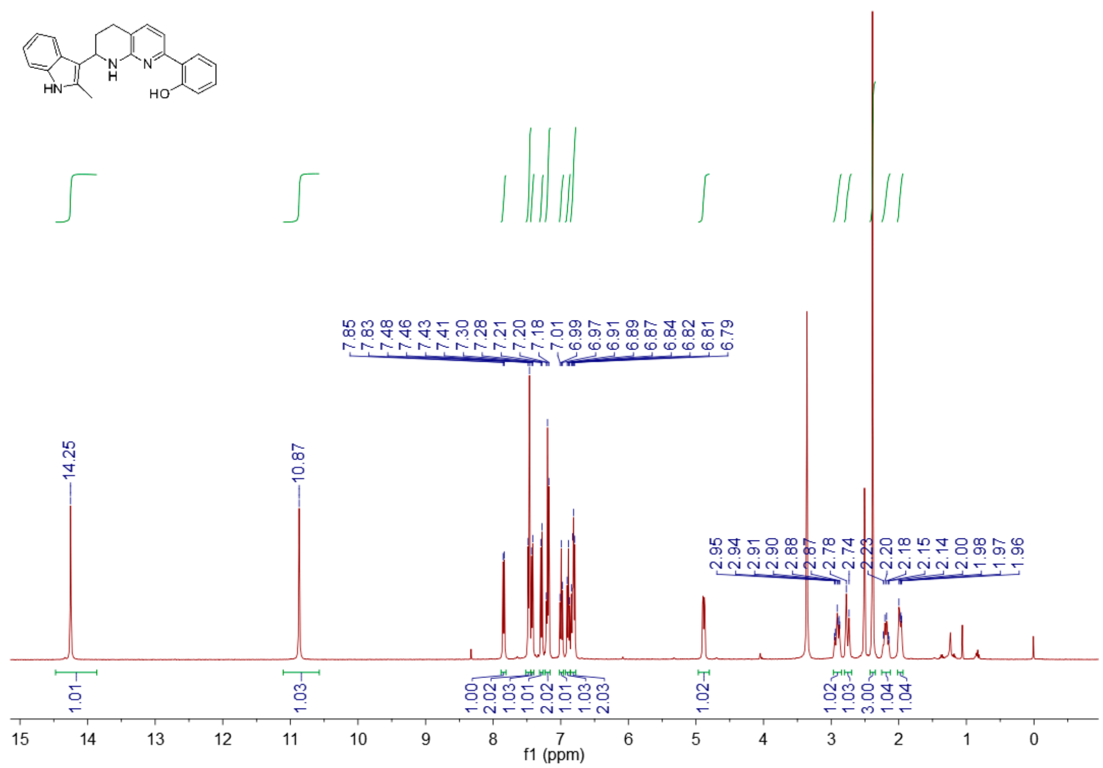

^{13}C-NMR spectrum of 3ha

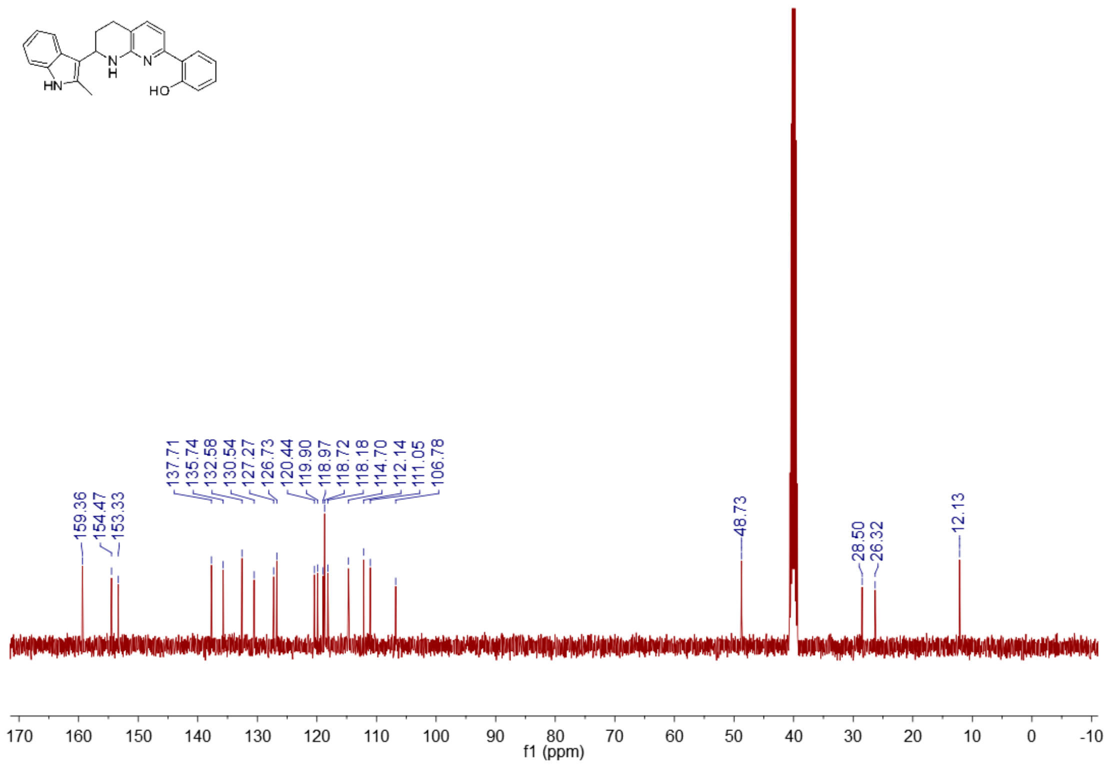

^{1}H-NMR spectrum of 3ia

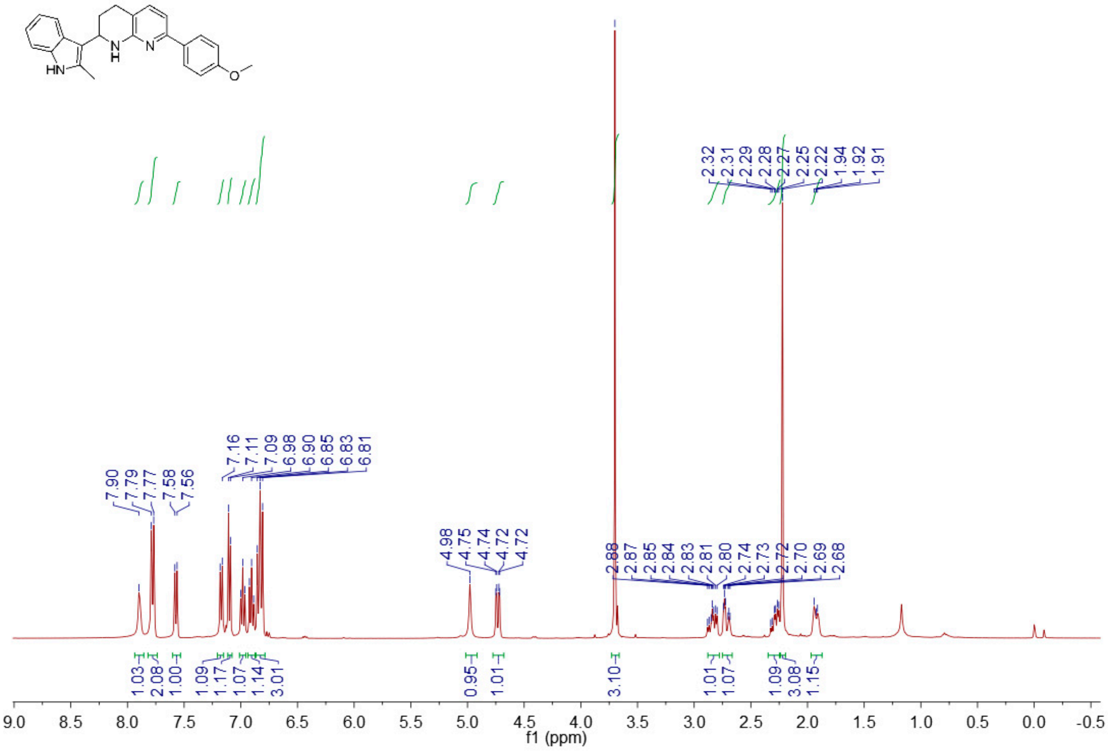

^{13}C-NMR spectrum of 3ia

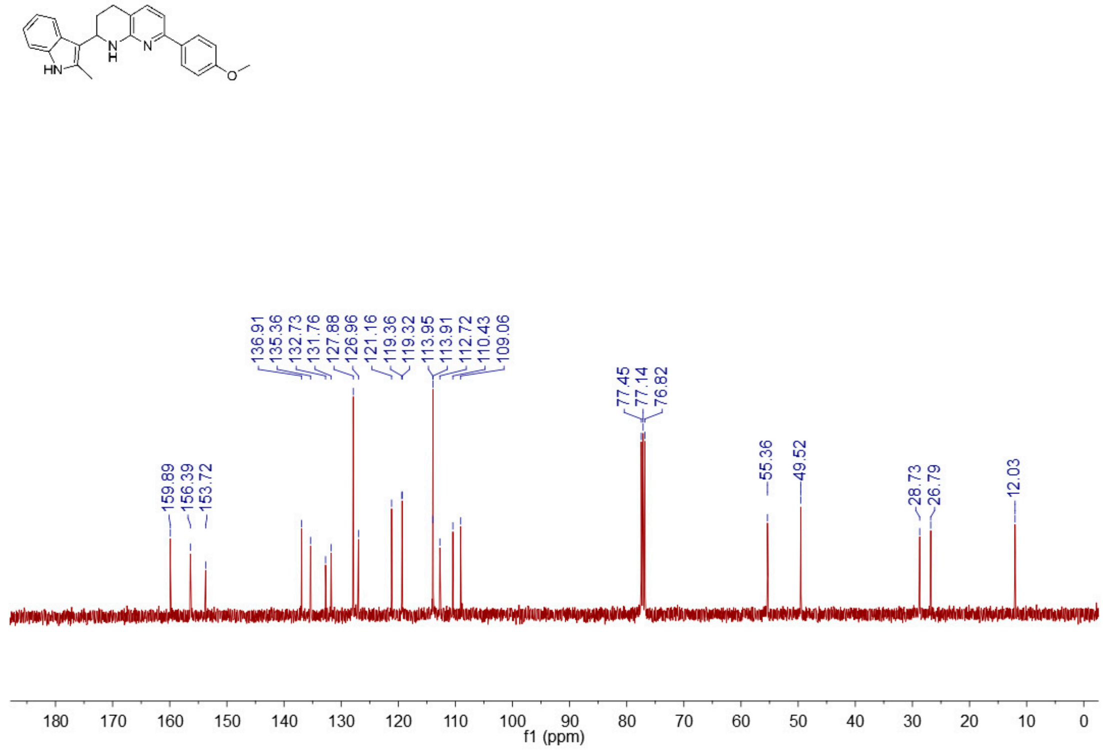

^{1}H-NMR spectrum of 3ja

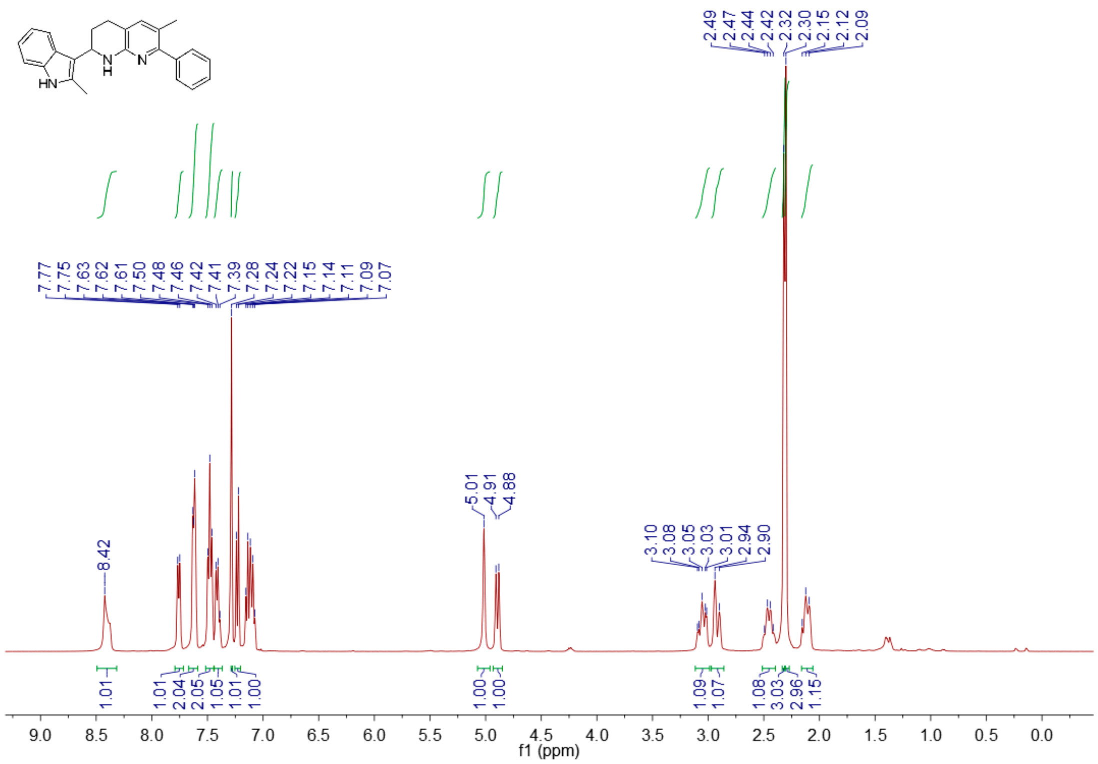

^{13}C-NMR spectrum of 3ja

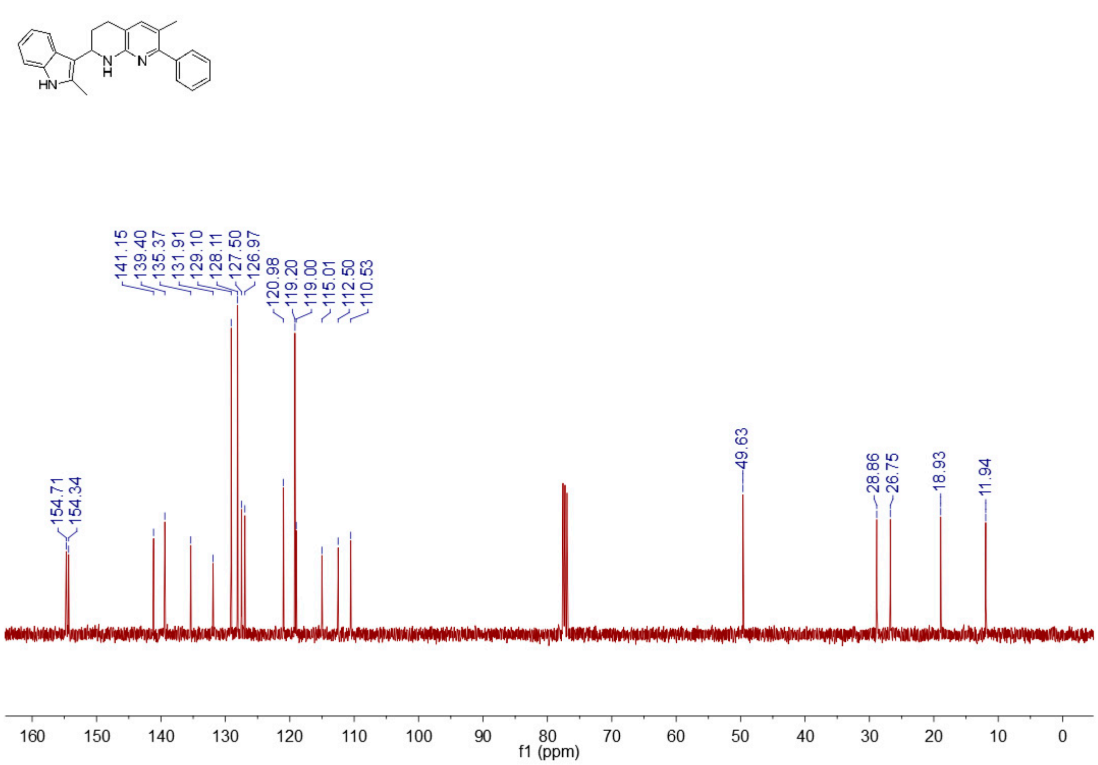

^{1}H-NMR spectrum of 3ka

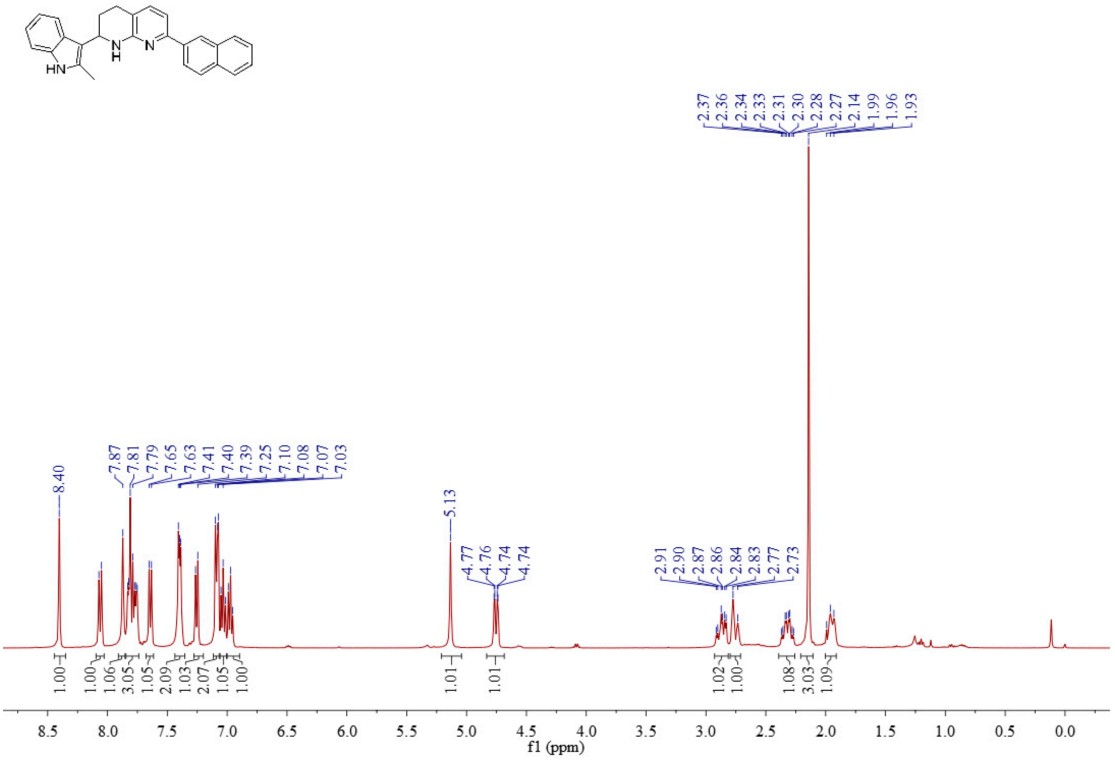

^{13}C-NMR spectrum of 3ka

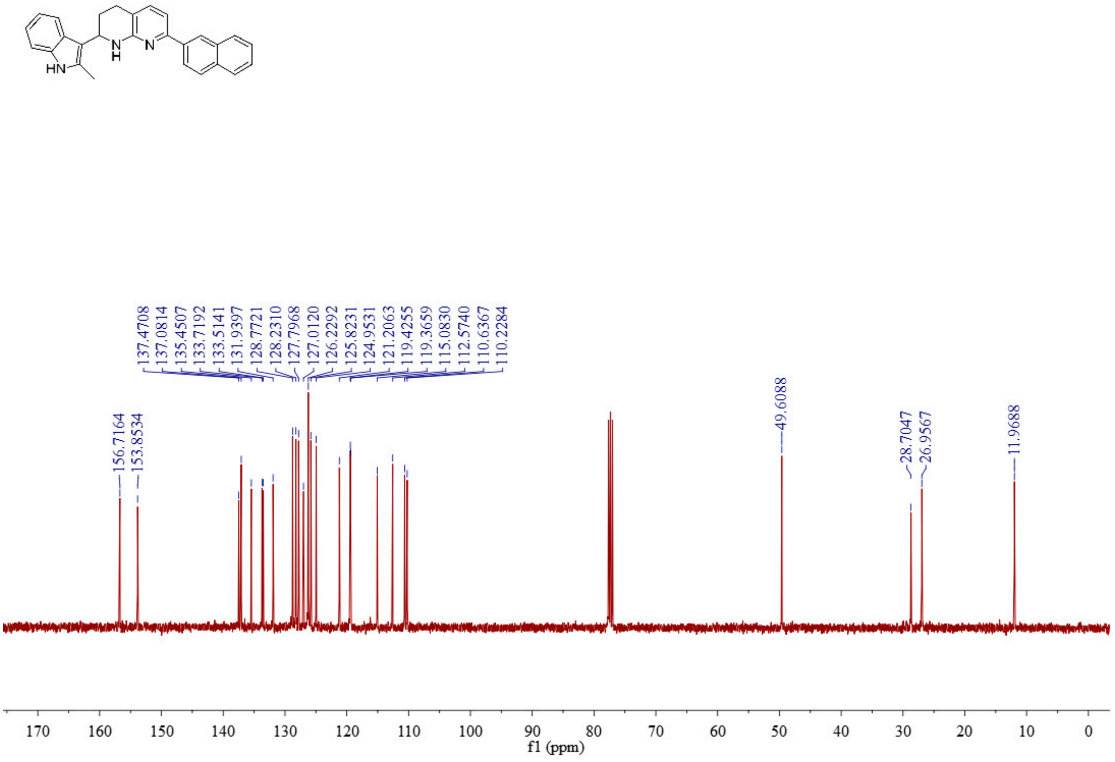

^{1}H-NMR spectrum of 3la

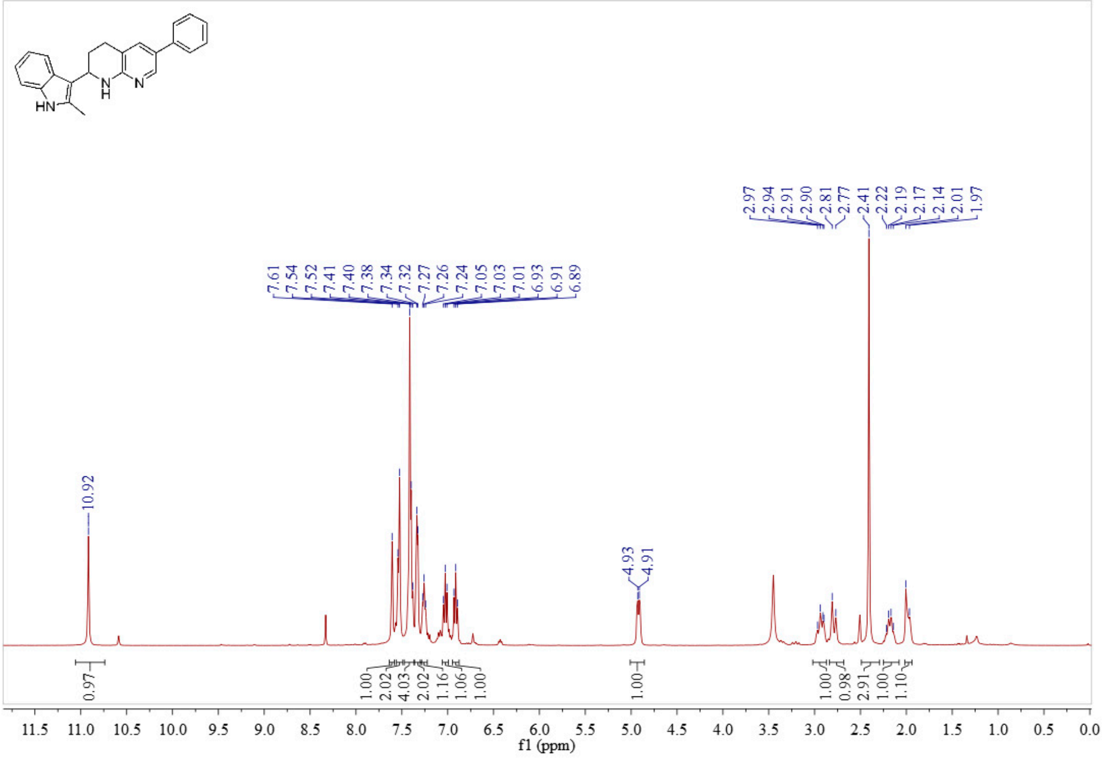

^{13}C-NMR spectrum of 3la

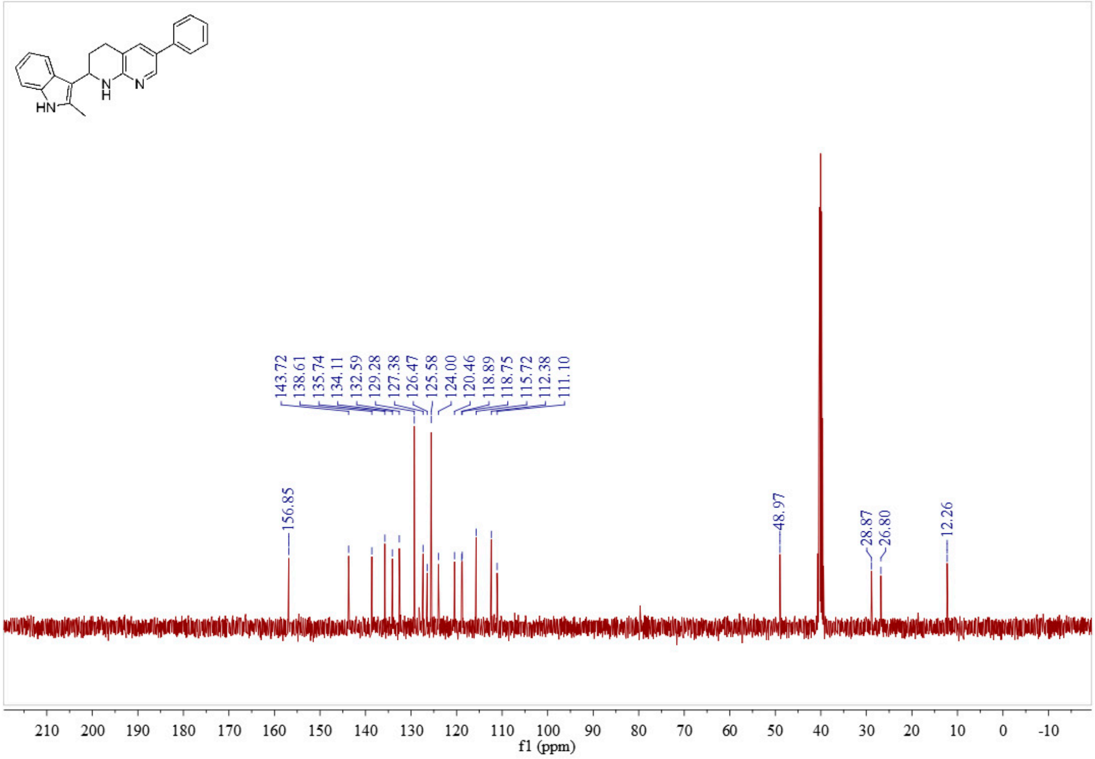

^{1}H-NMR spectrum of 3ma

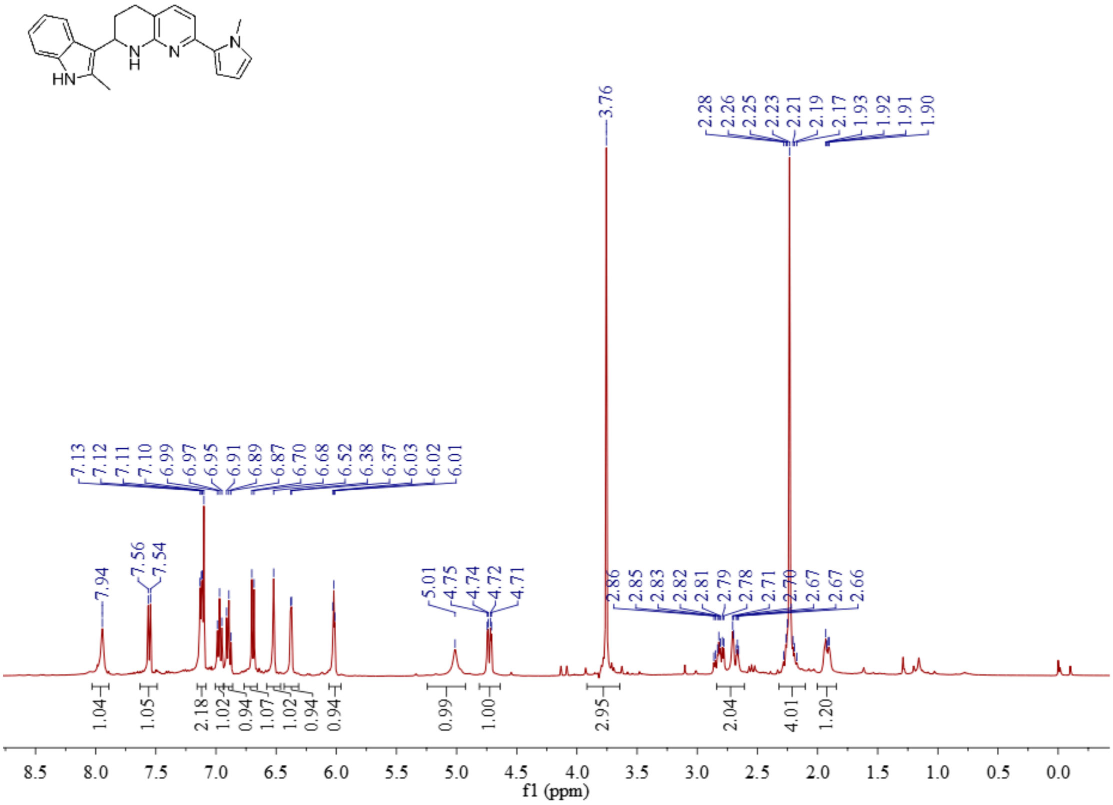

^{13}C-NMR spectrum of 3ma

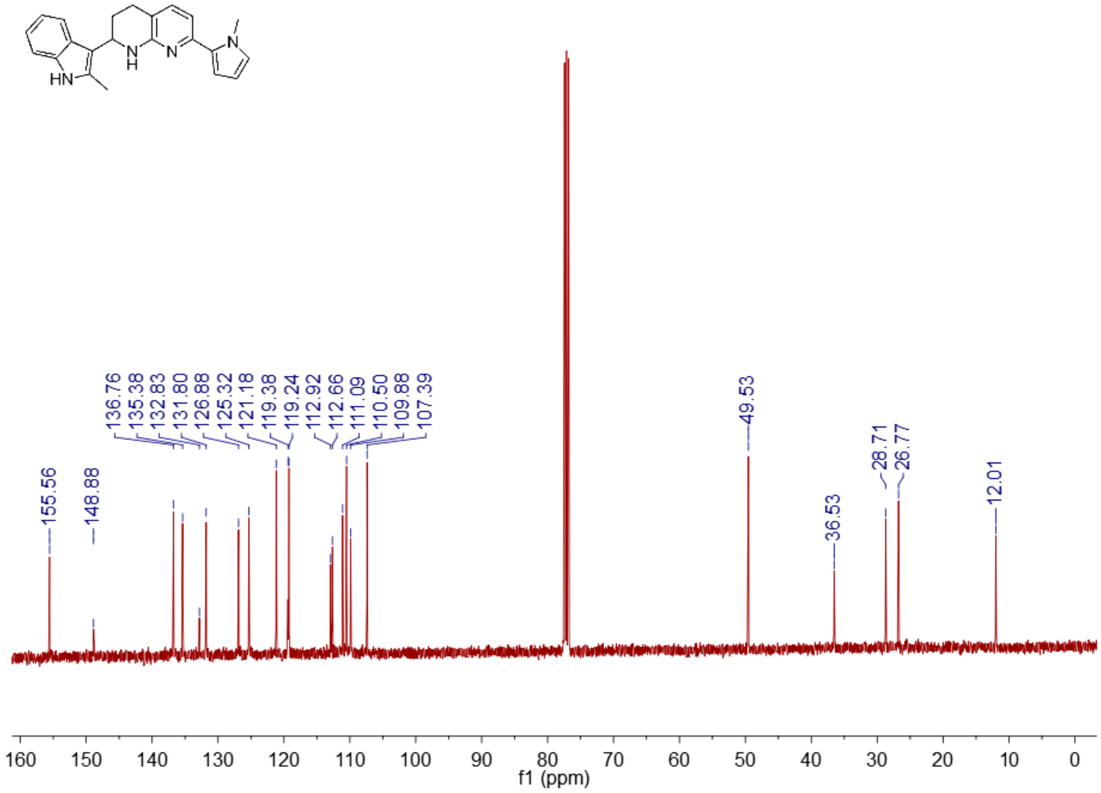

^{1}H-NMR spectrum of 3na

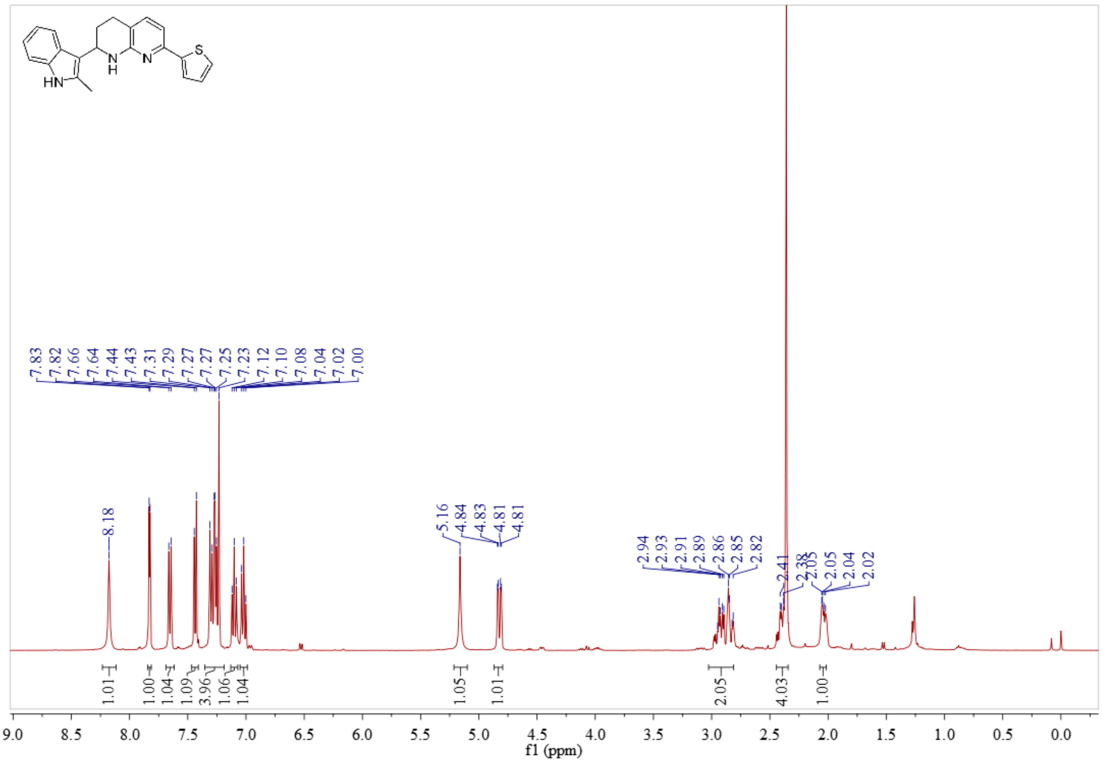

^{13}C-NMR spectrum of 3na

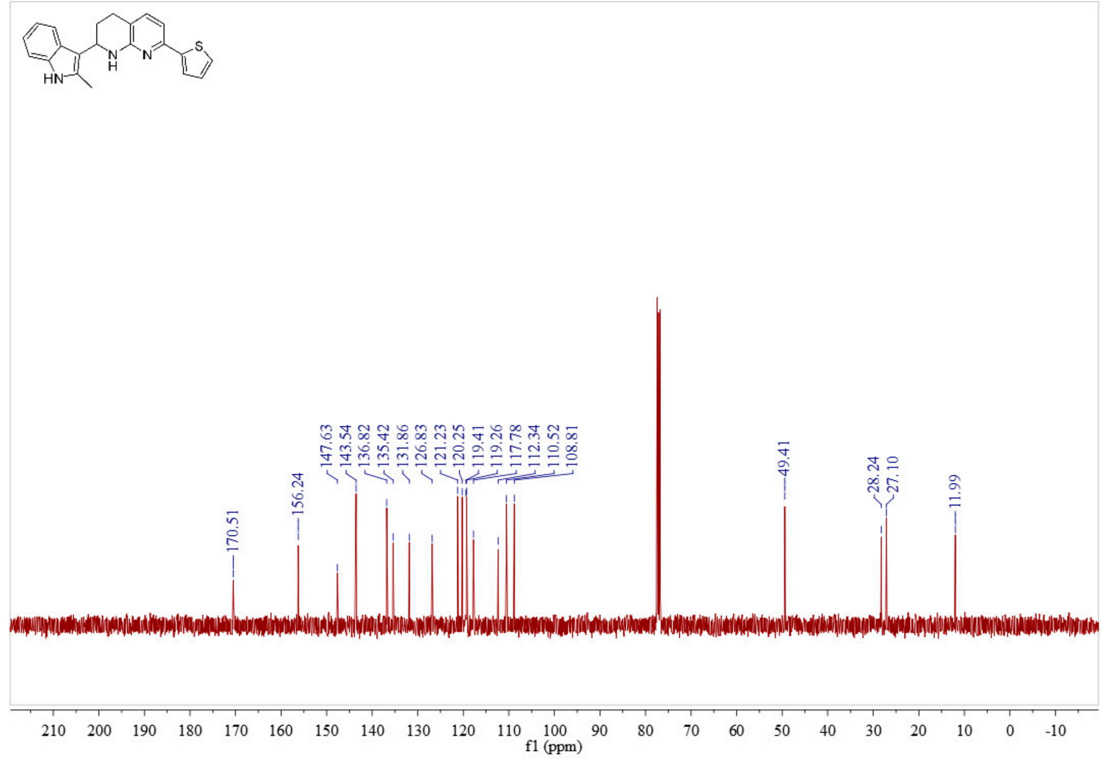

^{1}H-NMR spectrum of 3oa

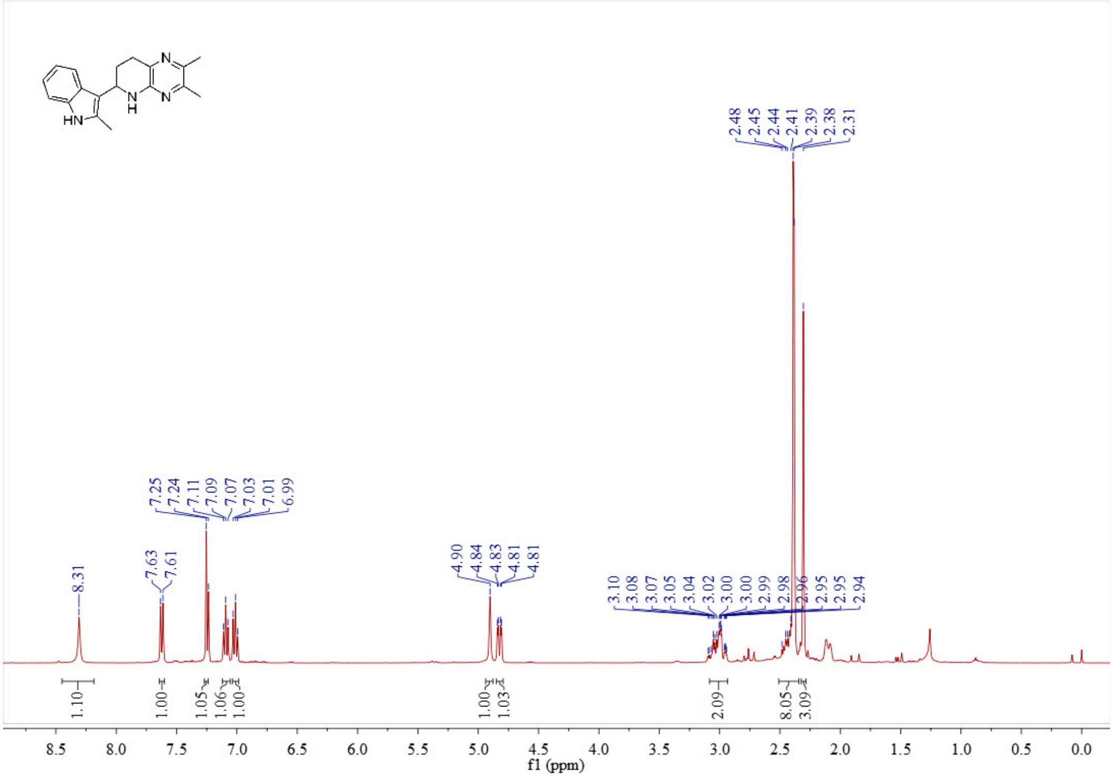

^{13}C-NMR spectrum of 3oa

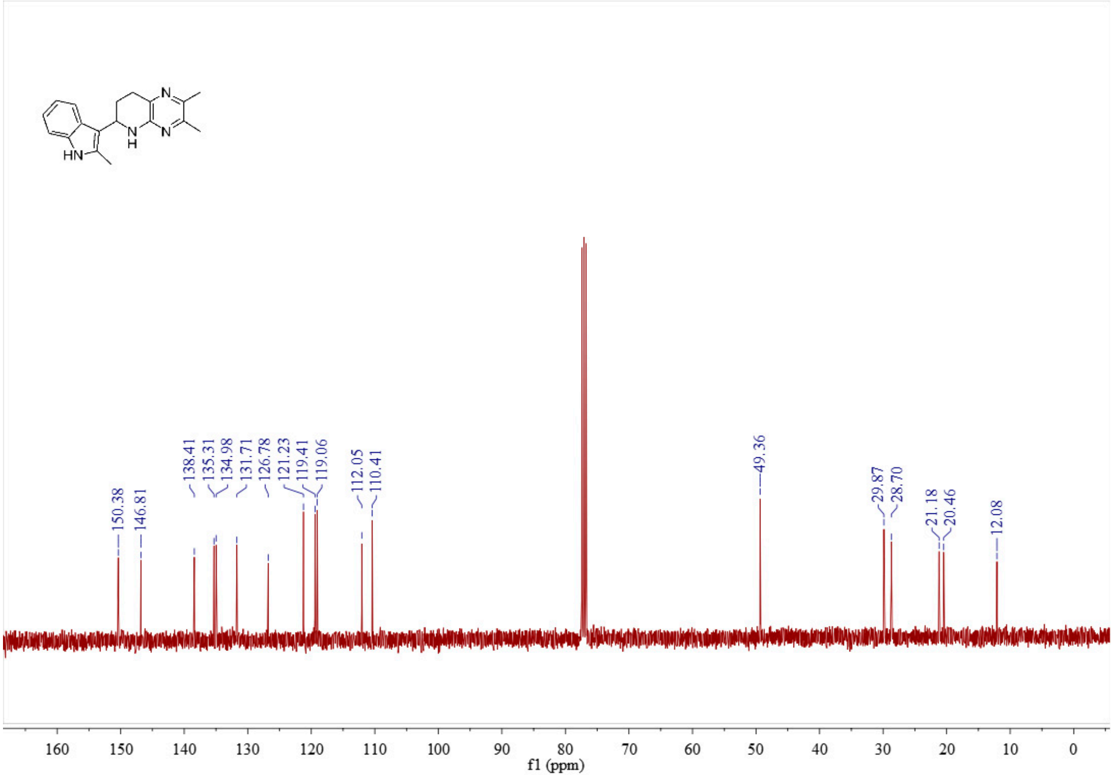

^{1}H-NMR spectrum of 3pa

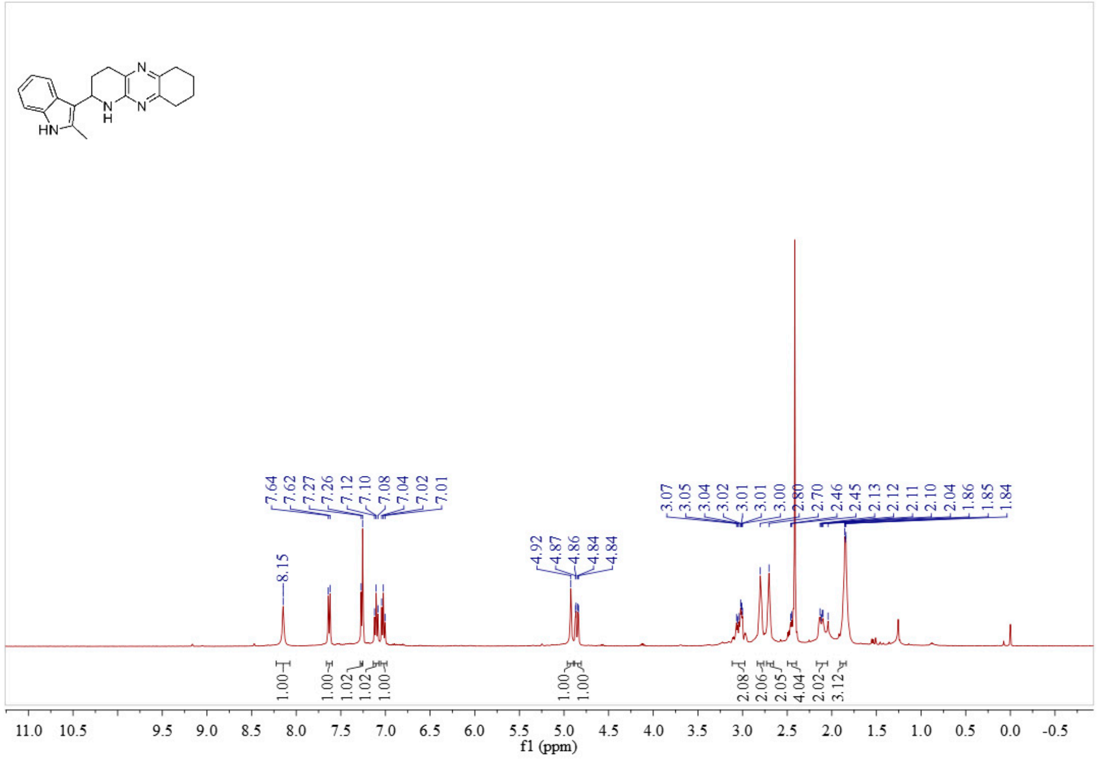

^{13}C-NMR spectrum of 3pa

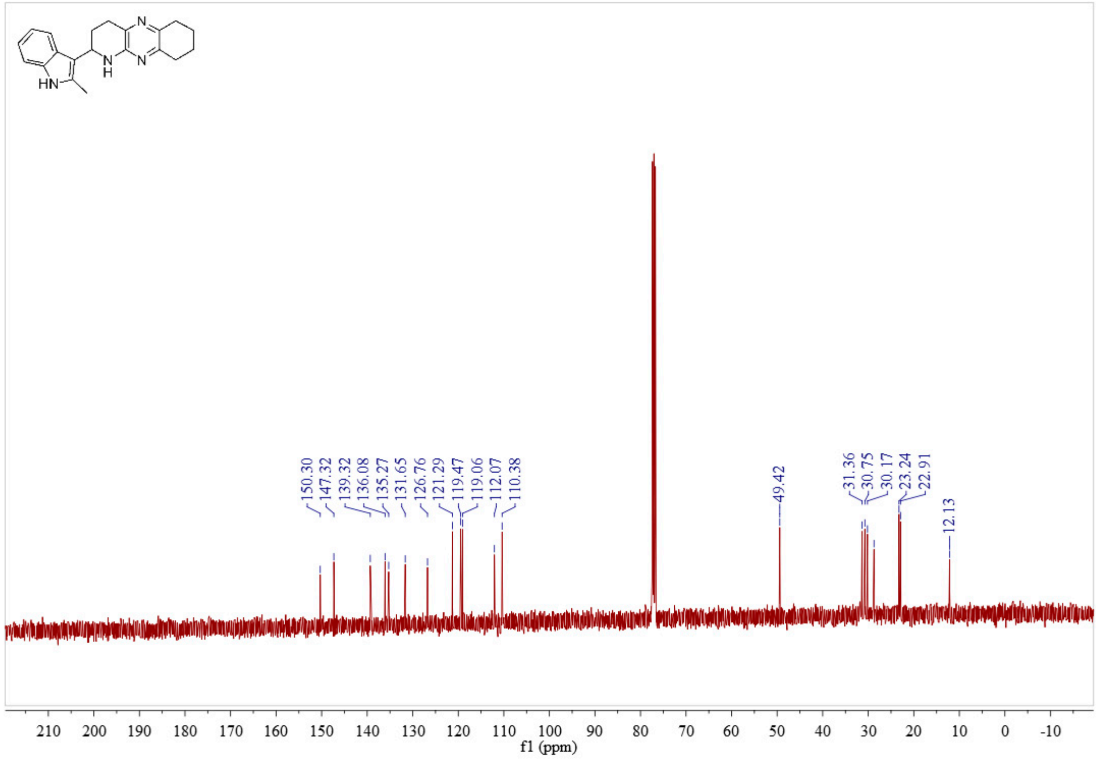

^{1}H-NMR spectrum of 3ab

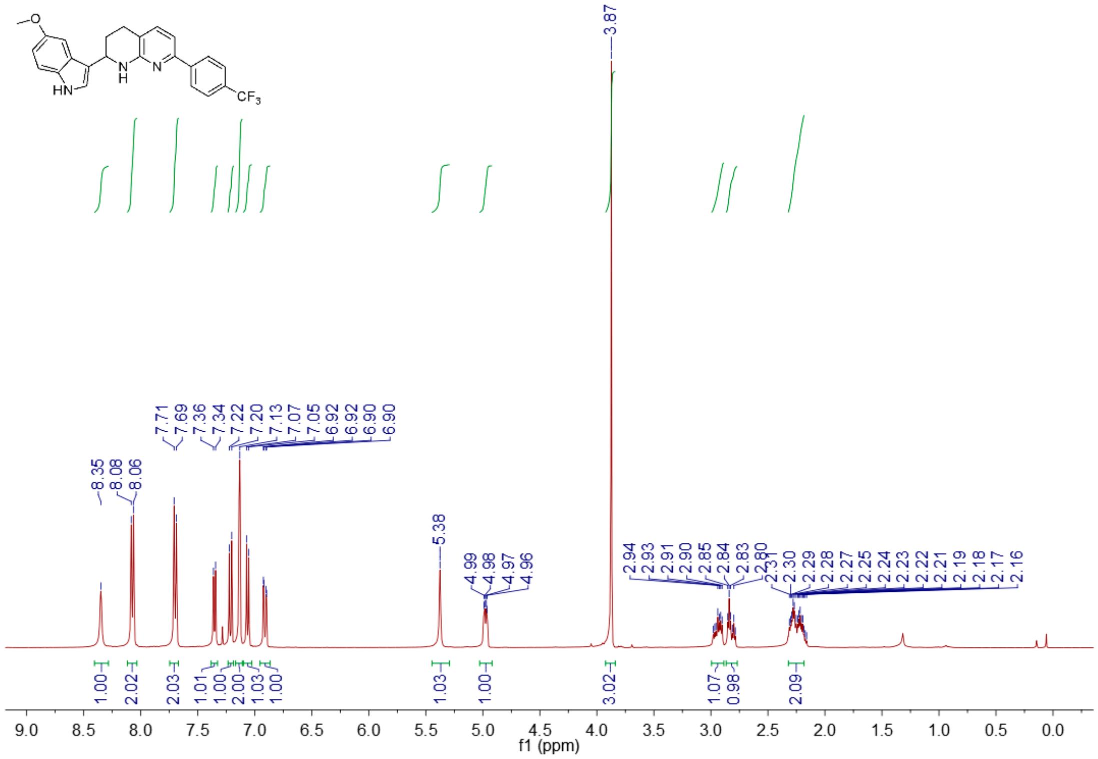

^{13}C-NMR spectrum of 3ab

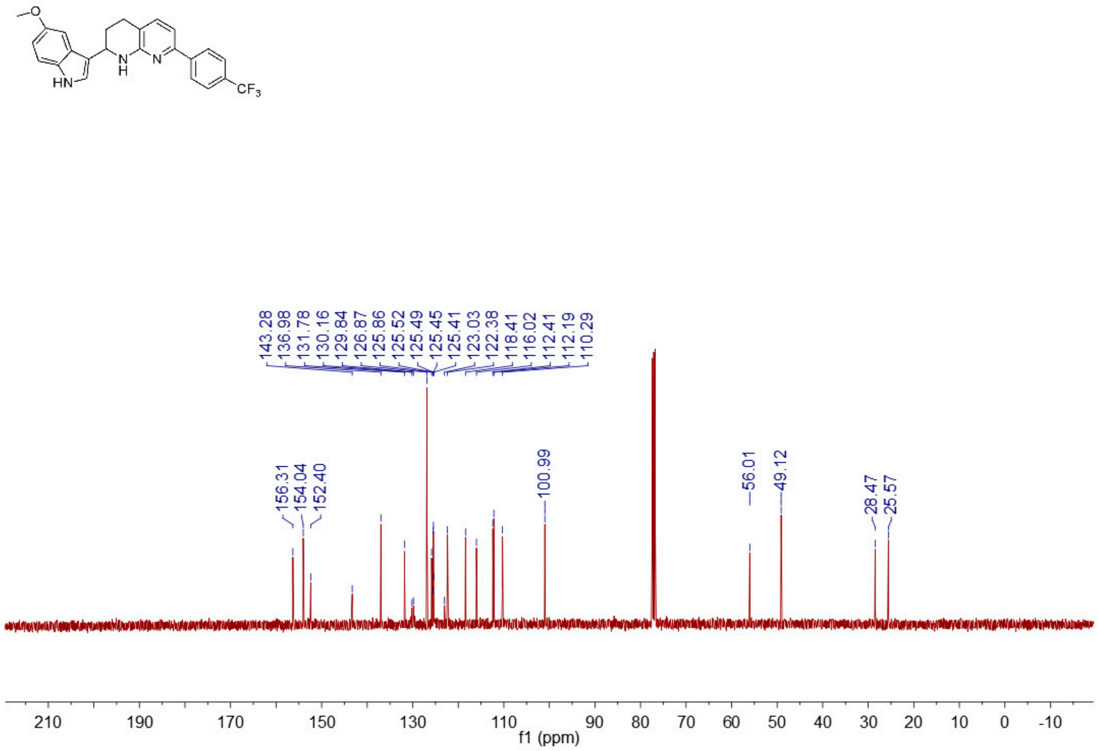

^{1}H-NMR spectrum of 3ac

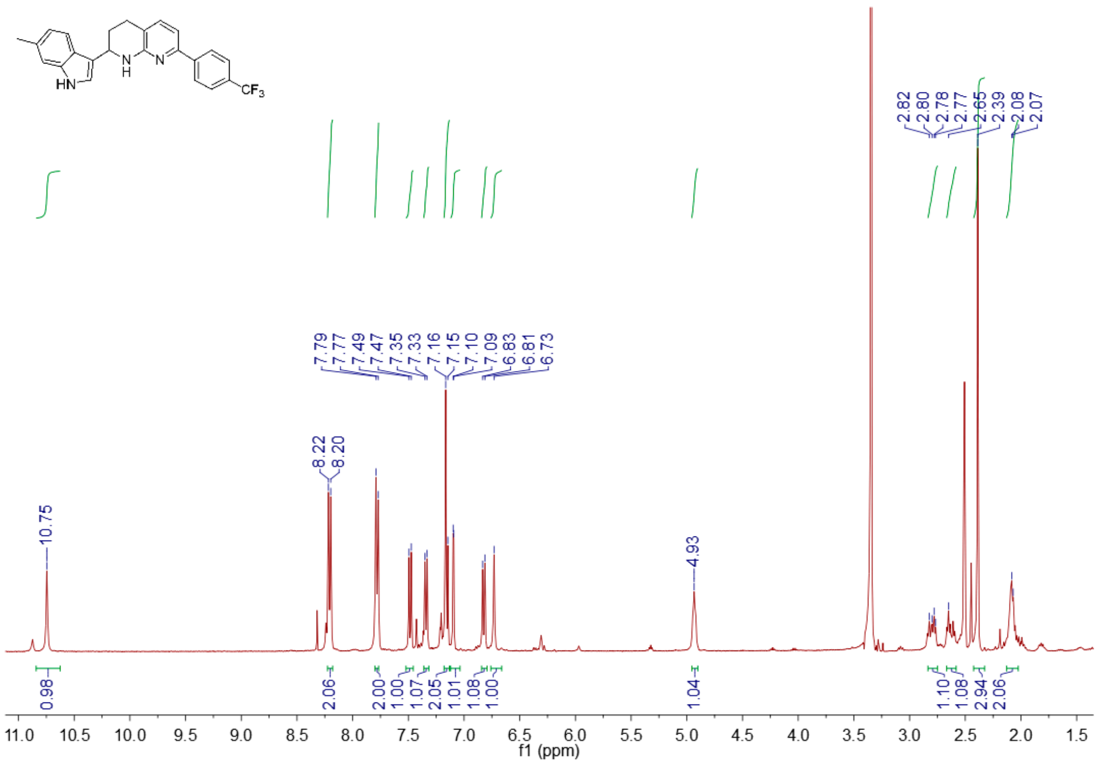

^{13}C-NMR spectrum of 3ac

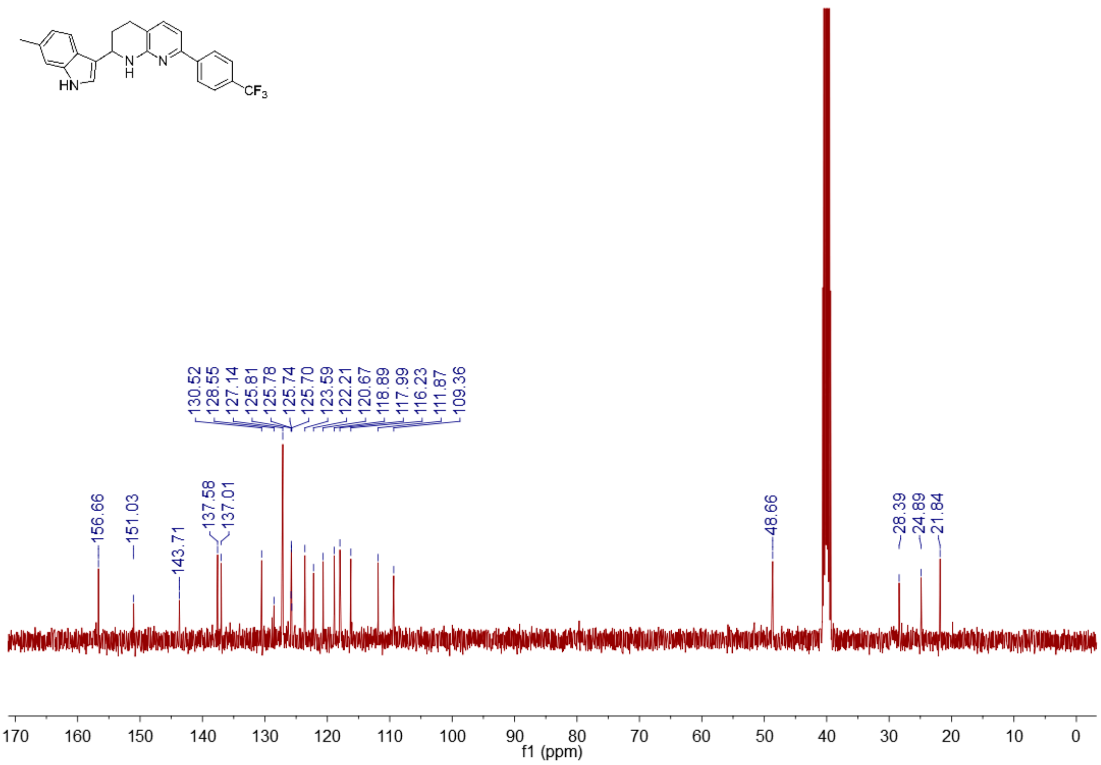

^{1}H-NMR spectrum of 3ad

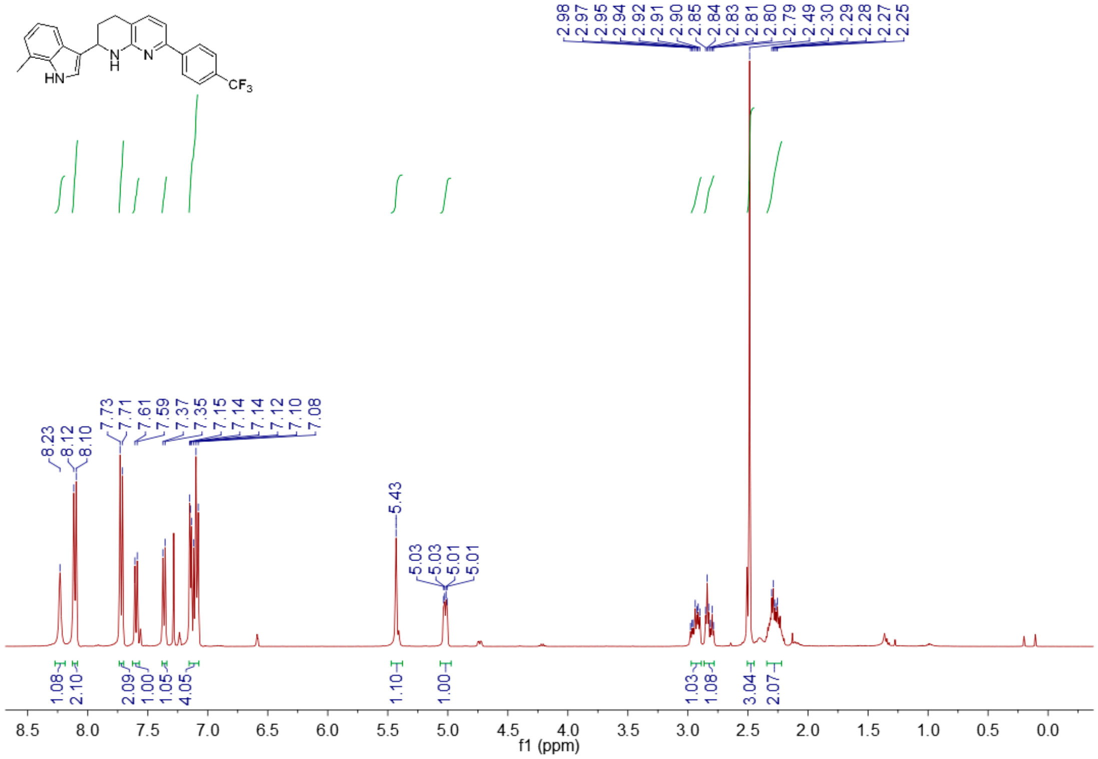

^{13}C-NMR spectrum of 3ad

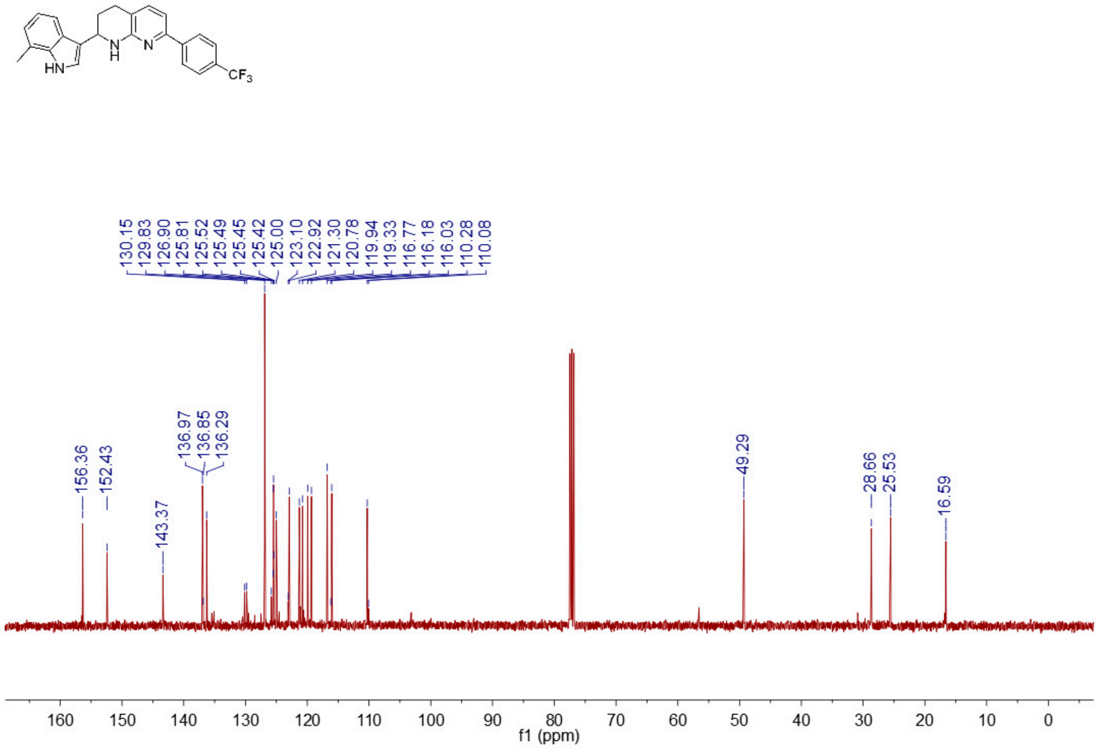

^{1}H-NMR spectrum of 3hb

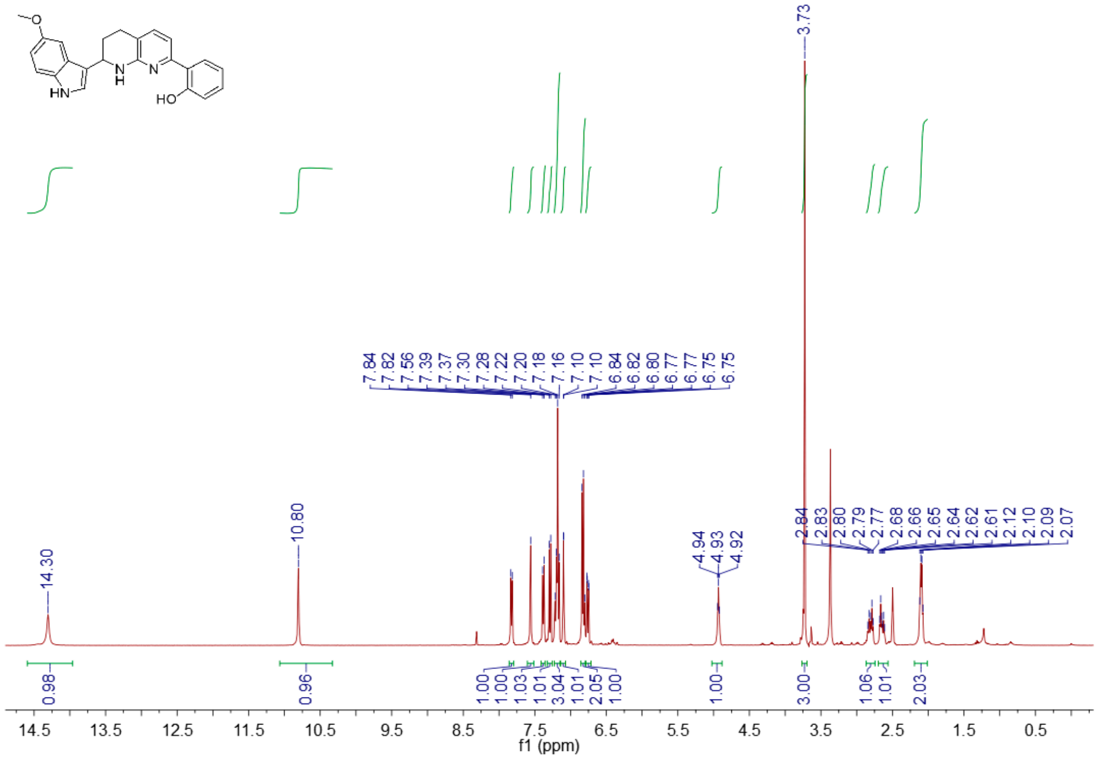

^{13}C-NMR spectrum of 3hb

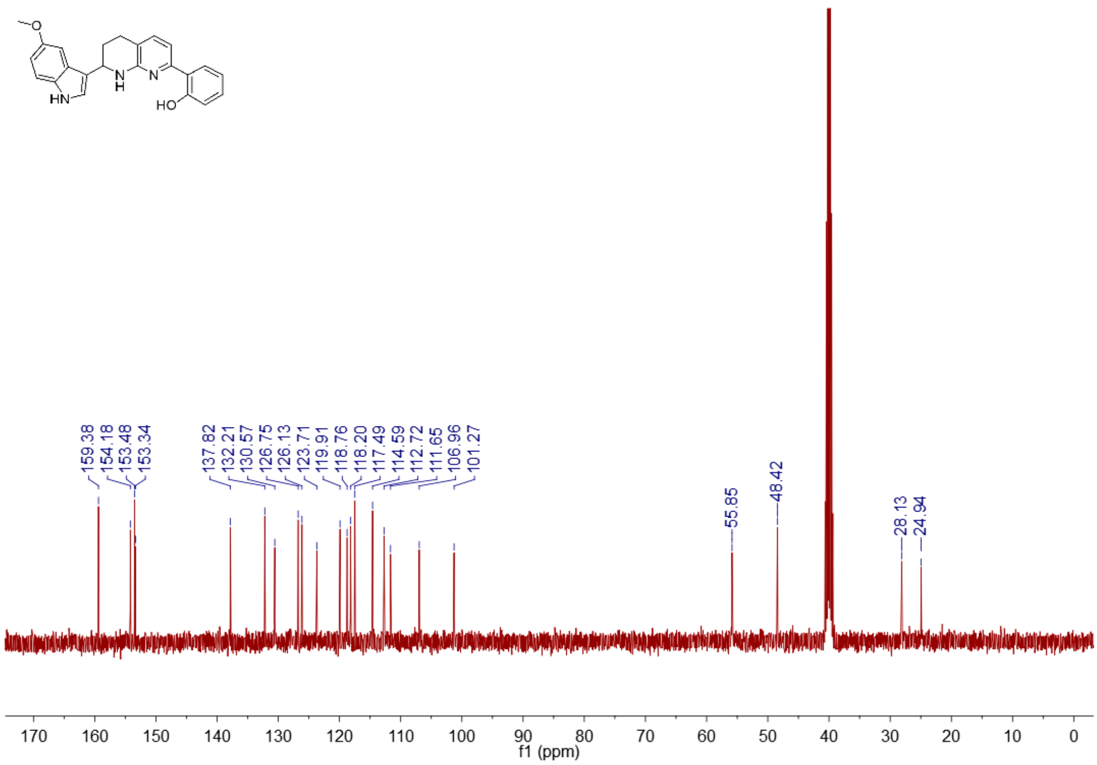

^{1}H-NMR spectrum of 3hc

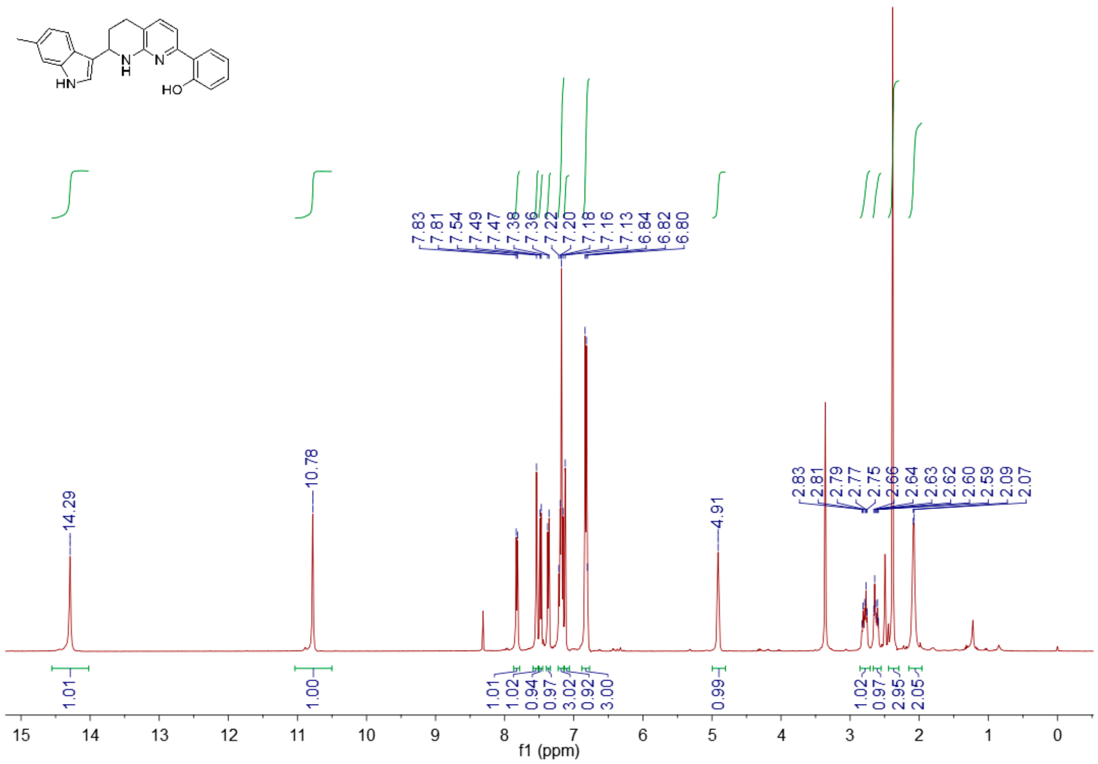

^{13}C-NMR spectrum of 3hc

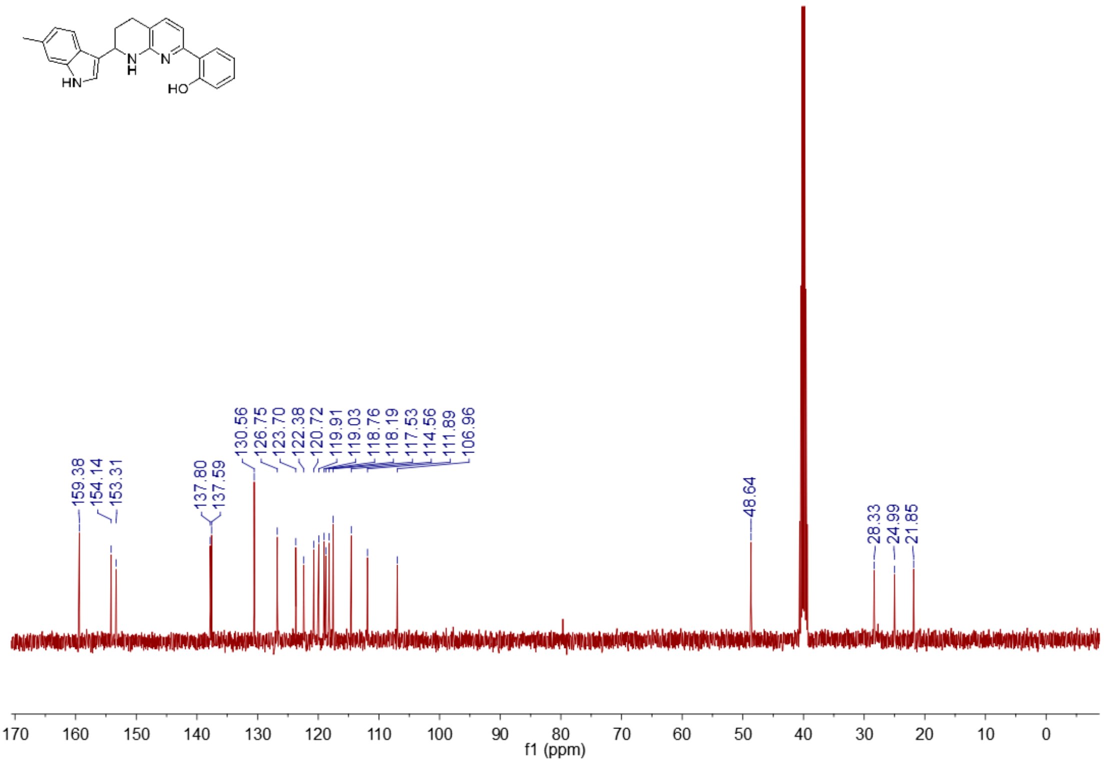

^{1}H-NMR spectrum of 3bd

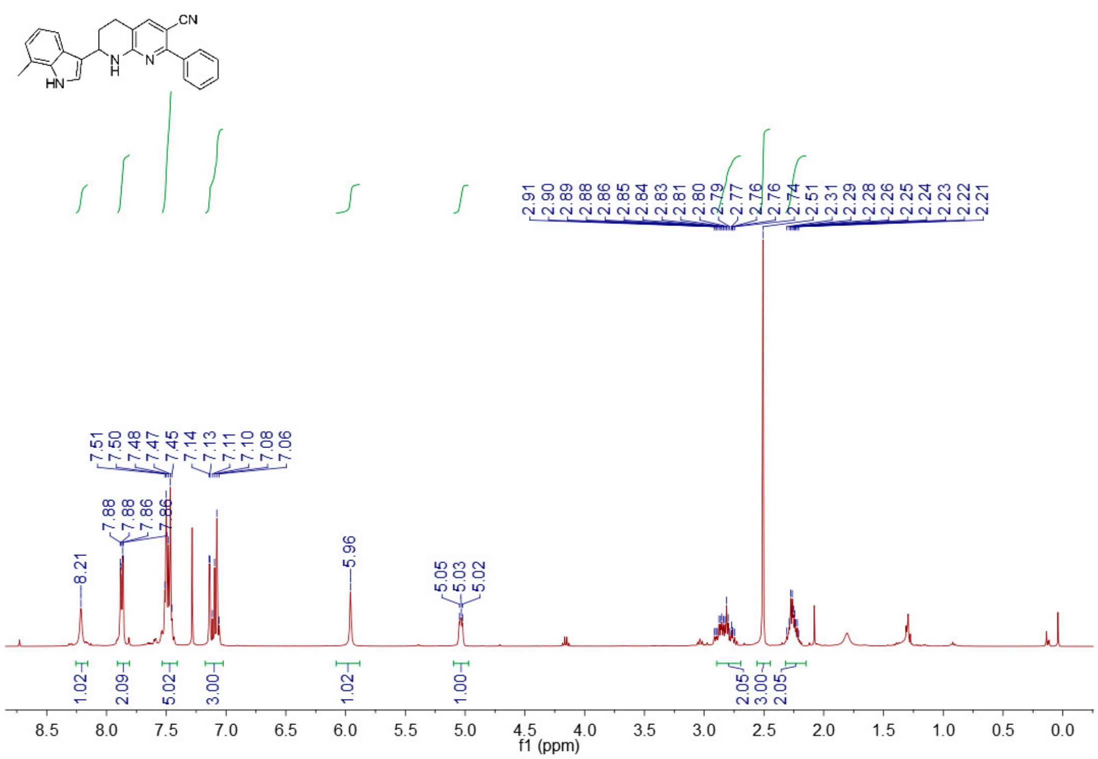

^{13}C-NMR spectrum of 3bd

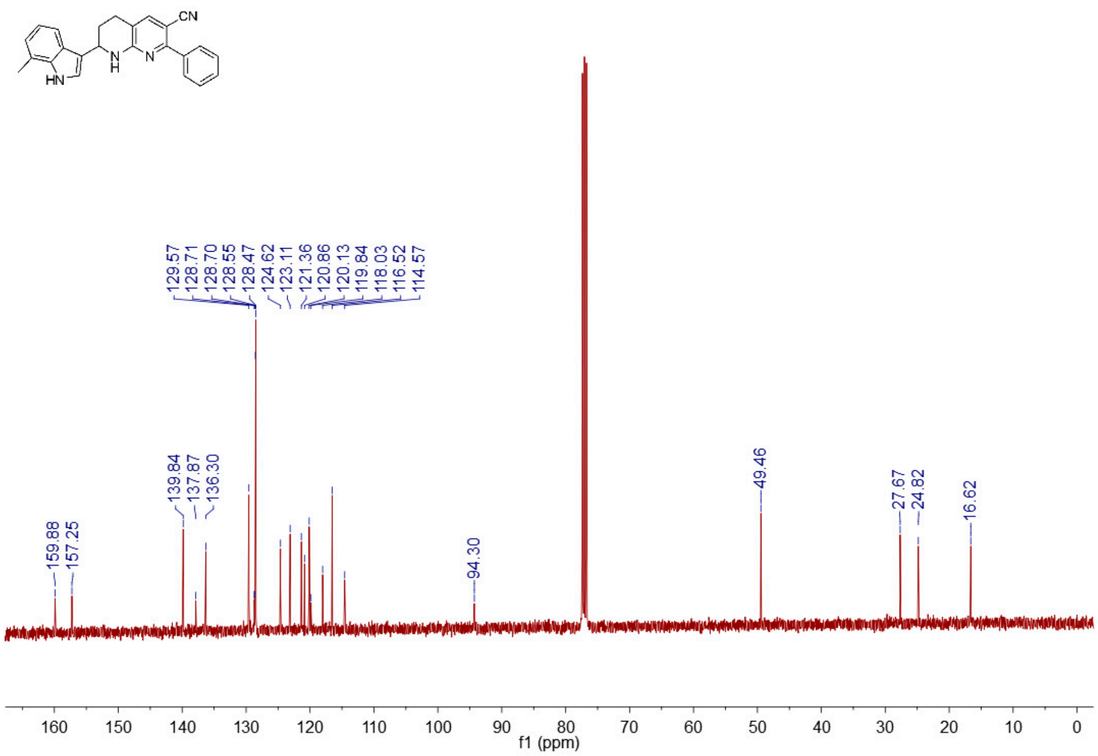

^{1}H-NMR spectrum of 3kb

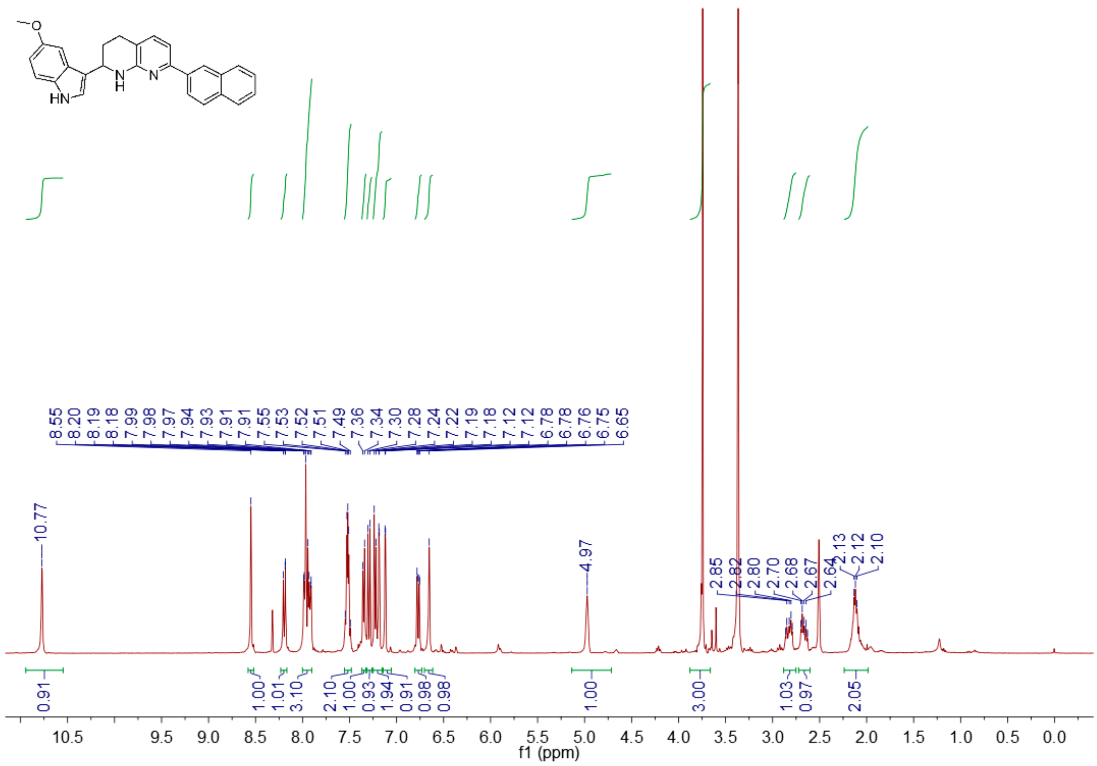

^{13}C-NMR spectrum of 3kb

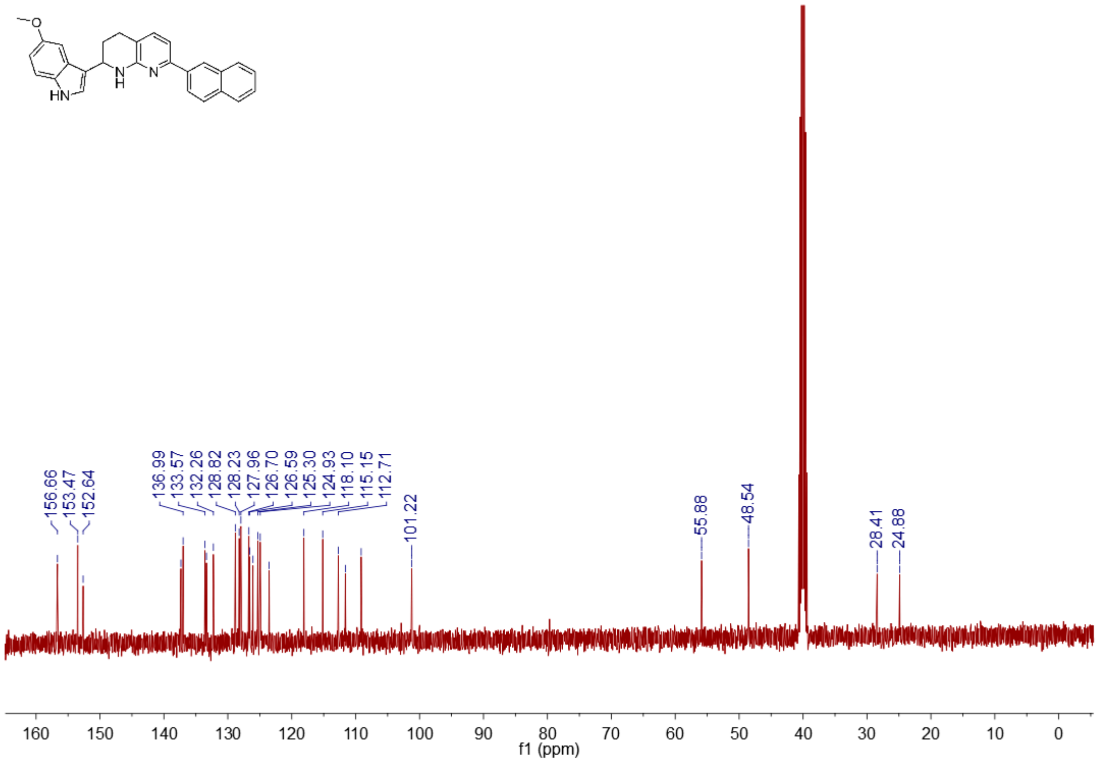

^{1}H-NMR spectrum of 3ae

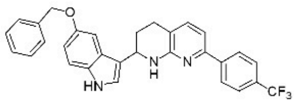

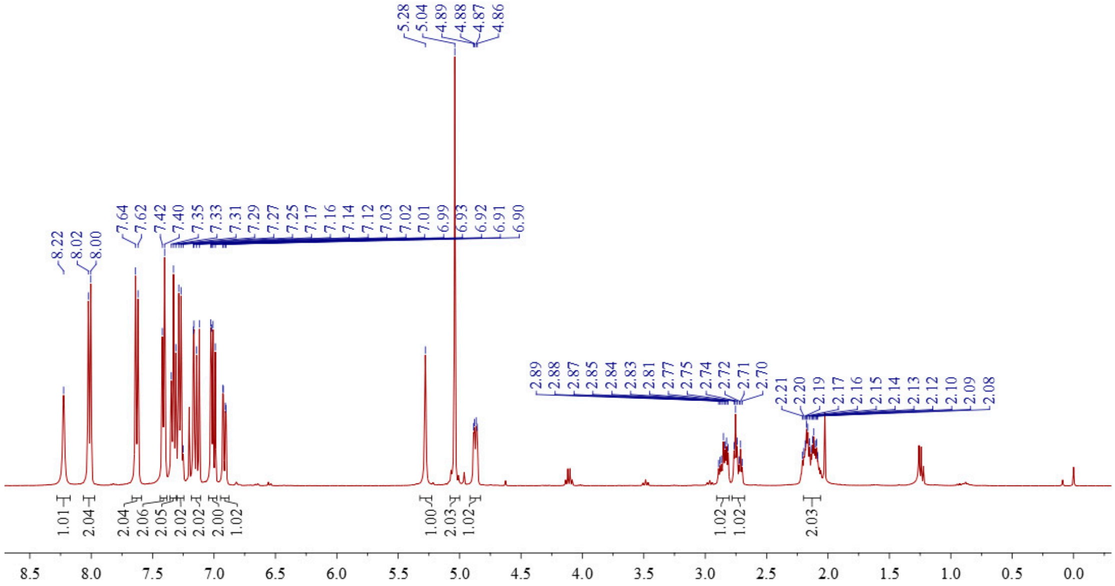

^{13}C-NMR spectrum of 3ae

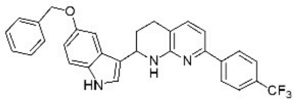

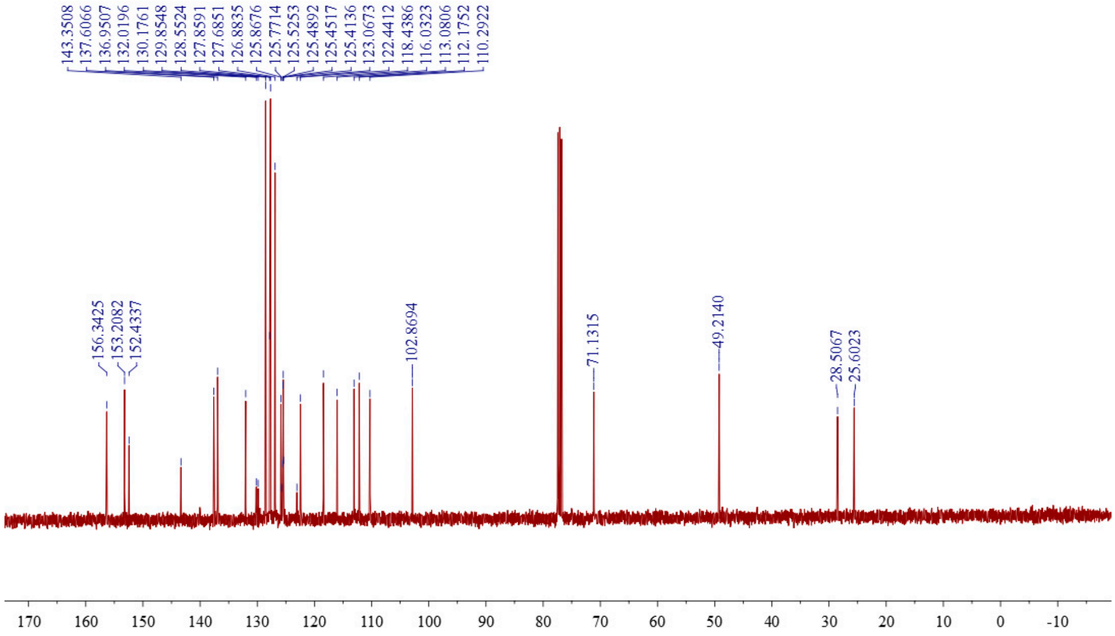

^{1}H-NMR spectrum of 3hf

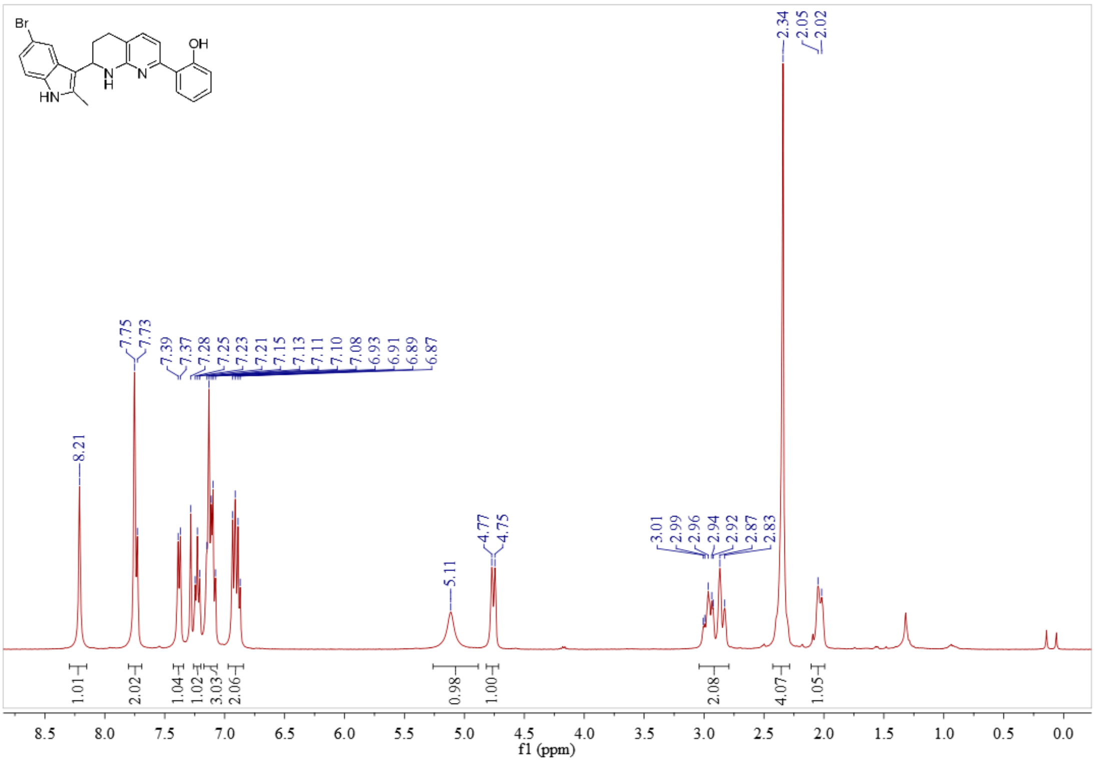

^{13}C-NMR spectrum of 3hf

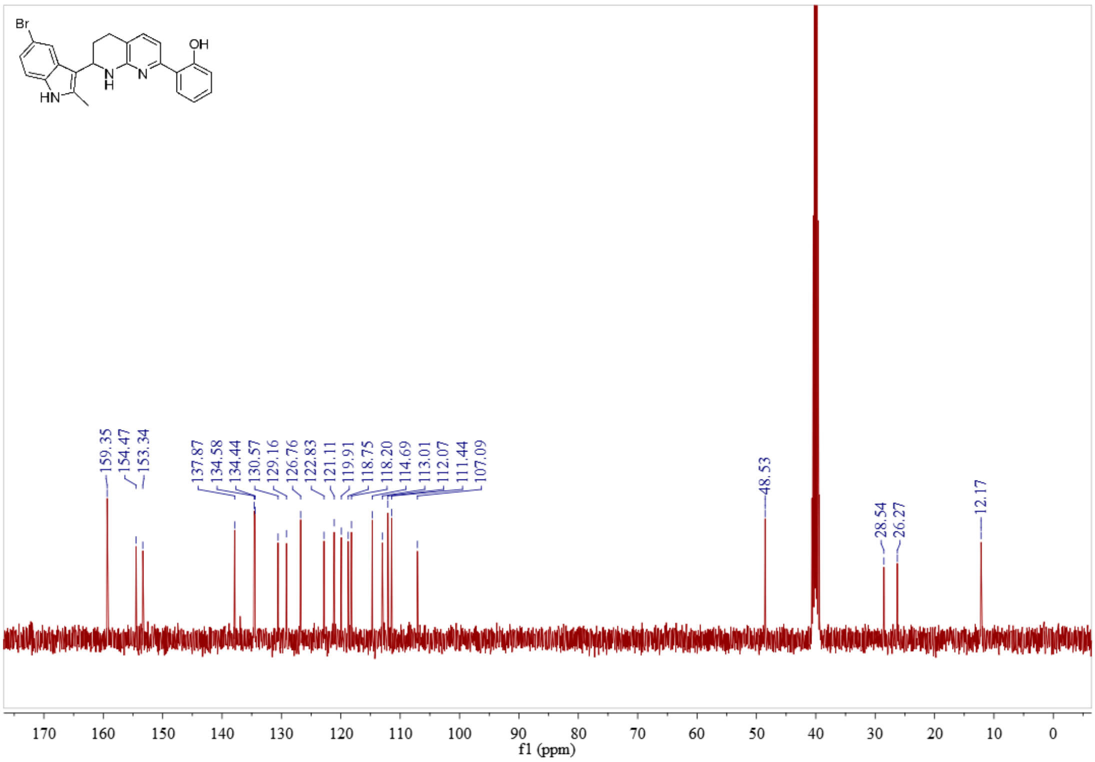

^{1}H-NMR spectrum of 3hg

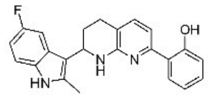

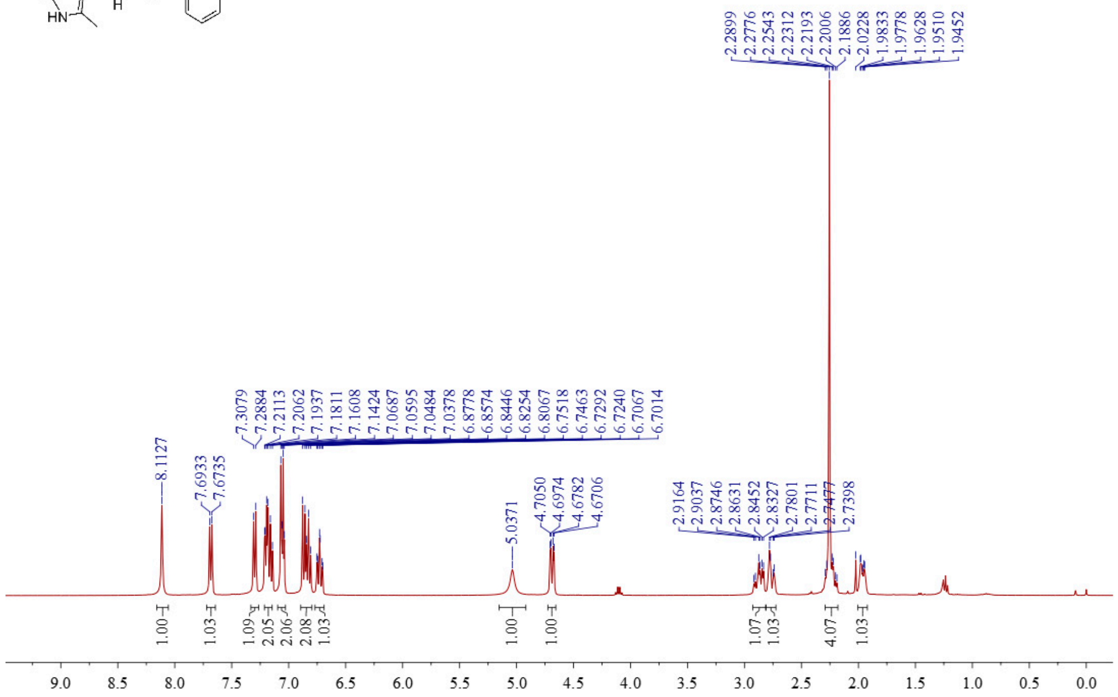

^{13}C-NMR spectrum of 3hg

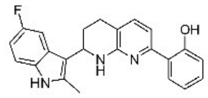

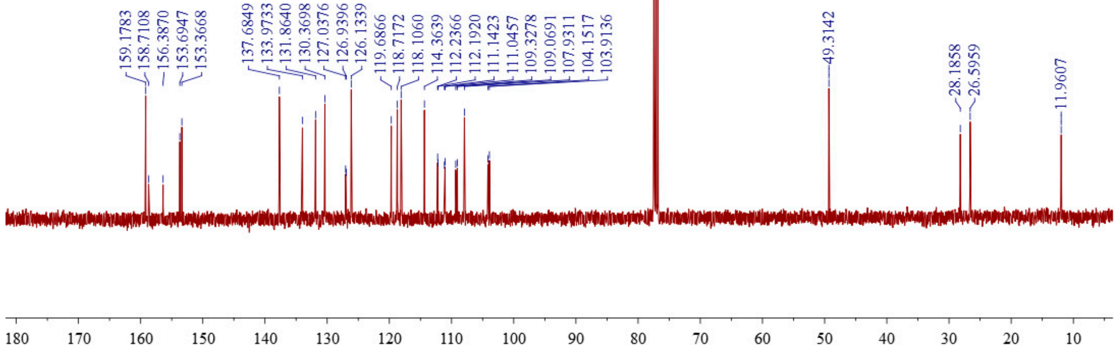

Supplement: Supplementary file 1 [file molecules-28-07886-s001.zip › molecules-2688925-supplementary.pdf]
